# Spatial Variation in Food Web Structures, Energy Flows, and System Attributes Along the Pearl River and Their Indications of Protection and Restoration

**DOI:** 10.1002/ece3.70991

**Published:** 2025-02-24

**Authors:** Sai Wang, Yong‐Duo Song, En‐Ni Wu, Tuan‐Tuan Wang, Muhammad Shahid Iqbal, Hui‐Long Ou, Jia Xie, Wen‐Tong Xia, Feng‐Juan Yang, Jie Feng, Shen‐Hao Wu, Yang Zhang, Cong‐Cong Jin, Zhuo‐Luo Ma, Hong‐Jin Zhang, Li‐Yong Miao, Kuan‐Song Wang

**Affiliations:** ^1^ State Key Laboratory of Marine Resource Utilization in South China Sea Hainan University Haikou China; ^2^ School of Marine Biology and Fisheries Hainan University Haikou China; ^3^ School of Ecology Hainan University Haikou China; ^4^ Ministry of Education Key Laboratory for Ecology of Tropical Islands, Key Laboratory of Tropical Animal and Plant Ecology of Hainan Province, College of Life Sciences Hainan Normal University Haikou China; ^5^ Guangdong Research Institute of Water Resources and Hydropower Guangzhou China; ^6^ Shenzhen Academy of Environmental Sciences Shenzhen China; ^7^ Shenzhen Guanghuiyuan Environment Water Co. Ltd. Shenzhen China; ^8^ China Water Resources Pearl River Planning Surveying & Designing Co. Ltd. Guangzhou China; ^9^ Hainan Qingxiao Environmental Testing Co. Ltd. Sanya China

**Keywords:** ecopath, ecotrophic efficiency, energy flow, food web, Lindeman spine, mixed trophic impact, system attribute, trophic level

## Abstract

River ecosystems are facing significant degradation from human activities, which impact both biotic (e.g., fish and invertebrates) and abiotic components (e.g., water and habitat). A comprehensive comparison of energy flow patterns and system attributes among river food webs under different levels of human interference is highly important for developing management strategies to protect river ecosystems. Along the subtropical Pearl River, six spatial zones, including agricultural, industrial, island, urban, factory, and estuarine areas, were chosen to construct the Ecopath models. The output results revealed that the highest trophic level of the Pearl River was 3.8–4.2, which was occupied by softshell turtles and piscivorous/carnivorous fish. The most diverse functional groups were found in island and estuarine zones due to their heterogeneous habitats (e.g., high submerged vegetation coverage and the transition area between fresh and brackish water). In contrast, the food web structure in the industrial zone was destroyed due to water pollution (e.g., sewage discharge) and habitat degradation. The increase in exotic species and the decrease in native top predators were two factors that result in the low efficiency of energy transmission. A series of trophic (e.g., Lindeman transfer efficiency and mixed predator–prey impacts), structural (e.g., keystoneness, omnivory, and Finn's path length), and theoretical (e.g., connectance and ascendency) indices revealed that the health and maturity of the Pearl River sections can be ranked as island > estuarine > agricultural > urban > factory > industrial zones. The food chains led by softshell turtles ( *Pelodiscus sinensis* ), piscivores (e.g., *Elopichthys bambusa* ), molluscivores (e.g., *Mylopharyngodon piceus* ), and herbivores (e.g., *Ctenopharyngodon idelus*) could be used to indicate the health and functioning of river ecosystems. Our results suggest that the ecological management of river ecosystems should focus more efforts on protecting original habitats (e.g., the island zone with fish feeding/spawning grounds), monitoring bioindicators with keystone trophic impacts in the food web, and evaluating the food chains that play important roles in upward energy transmission.

## Graphical abstract

The undisturbed island zone, which provided essential feeding/spawning habitats, should be protected to maintain river health. The food web structure in the industrial zone was collapsed due to water pollution and habitat degradation. The appearance of softshell turtles and herbivorous/molluscivorous fish can be used to indicate the integrity of the food web structure.

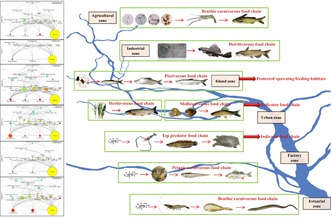

## 1 Introduction

Longitudinal variation in fluvial habitat characteristics and water environment determines regional differences in biological communities and food web structures along the river continuum (Vannote et al. 1980). Currently, river landscapes are continuously modified by humans to maximize the exploitation of water resources to meet the needs of social and economic development (Tram et al. 2022). How rivers are affected by anthropogenic disturbances includes land use change (e.g., urbanization and industrialization), hydraulic engineering, water pollution, deforestation, overfishing, invasive species, and climate change (Calizza et al. 2012). In large river basins with dense river populations, especially those located in fast‐growing urban zones, direct and indirect external disturbances may not only destroy the original natural environment but also pose inordinate threats to fluvial organisms, leading to population decline, a decrease in community diversity (Guzelj et al. 2020), and even the collapse of aquatic food webs (Wang et al. 2018a).

Among the multiple human‐induced stressors, land use changes, e.g., agriculturalization, urbanization, and industrialization, are the prevalent causes leading to habitat degradation in both river channels and riparian zones (Tang et al. 2025; Wang et al. 2024c). The negative impacts of habitat degradation on river ecosystems can be physical (e.g., homogeneous substrates and flow regimes), chemical (e.g., contaminant inputs and toxic effects), or biological (e.g., limitations on food resources) (Reid et al. 2019; Wang et al. 2022). For example, Hette‐Tronquart et al. (2018) investigated community‐level metrics at sites with contrasting land uses in the Seine River in France and reported that urbanization at the local scale affected the fish community composition. Price et al. (2019) reported that in Zagreb (Croatia), macroinvertebrates in urbanized streams presented lower trophic redundancy than did their counterparts in woodland and agricultural streams. Wang et al. (2020a) suggested that the simplified food web structures observed in the lower East River should be attributed to urbanization, which resulted in the limitation of available resources (e.g., feeding and reproduction grounds). Moreover, the loss of functional groups that play keystone roles in trophic control (e.g., top‐down and bottom‐up effects), such as top fish predators needing multiyear sexual maturity and spawning migration, may lead to the collapse of the food web structure (Wang et al. 2024a, 2021a, 2024b).

A better understanding of how habitat degradation affects community structure and food web attributes is essential in aquatic science and has implications for the sustainable management of riverine ecosystems (Brett et al. 2017; Wilson et al. 2016). Current studies have focused on the influence of land use change on population and community diversity; however, both species‐ and community‐level research could reflect only partial aspects of the ecosystem, with evidence on trophic networks and energy flows from the perspective of the food web still limited (Wang et al. 2020a, 2020b). Food web properties (e.g., energy transfer efficiency, trophic interactions, and omnivory) are important factors reflecting the comprehensive human disturbance in river ecosystems, although monitoring and identifying key problems from a system perspective are difficult (Wang et al. 2022). The lack of data is a barrier to the development of basin‐scale protection and management strategies. Thus, establishing an ecological model can quantify the various indicators of the ecosystem and scientifically evaluate the impact of the external environment on the ecosystem (Chovanec et al. 2015; Wang et al. 2023a, 2023b).

The ecopath model, also known as the ecological channel model, is an energy balance model that directly constructs the structure of aquatic ecosystem, describes energy flow, and determines ecological parameters via nutrient dynamics (Pauly et al. 2000); its basic functions include quantifying and synthetically analyzing the process of nutrient flow in the ecosystem, clarifying the distribution and circulation of energy flow and the efficiency of energy flow among different trophic levels, and determining the scale, stability, and maturity of the system (Christensen and Walters 2004). The model was created in 1984 and was followed by multiple ecological theories. After years of development, the model has become a key tool for research on aquatic ecosystems, such as oceans, bays, lagoons, rivers, streams, wetlands, and reservoirs; specifically, it has become an important method for studying the structure and function of food webs (Christensen and Walters 2004).

Information on the trophic networks and energy flows of large rivers is scarce, and the spatial variations in ecosystem attributes are poorly understood (Chovanec et al. 2015; Wang et al. 2020a, 2019a). Studying the influence of anthropogenic disturbances on fluvial food web structure and attributes is important for regional conservation and ecosystem management, especially for rivers in the tropics and subtropics, where there is extreme biodiversity (Townsend and Riley 2010; Wang et al. 2018b). In this context, the main problems to be solved in this paper are (1) spatial variation in food web structures, energy flows, and system attributes along a disturbed river, and (2) the degradation mechanism of the river ecosystem under different degrees of human disturbance. By solving these problems, we can learn and predict how human interference affects the environment (e.g., water quality and habitat characteristics), communities (e.g., fish and invertebrates), and predator–prey interactions between food chains and among food webs. These results are conducive to the conservation and restoration of river ecosystems.

## 2 Materials and Methods

### 2.1 Study Area

**FIGURE 1:**
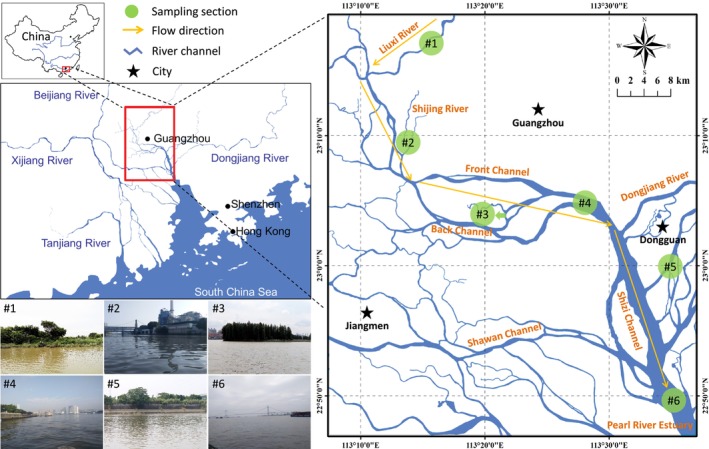
Six sampling sections along the main channel of the Pearl River. River sections #1 to #6: Agricultural, industrial, island, urban, factory, and estuarine zones, respectively.

The Pearl River is the second‐largest river in China in terms of annual runoff (3.3 × 10^11^ m^3^), exceeded only by the Yangtze River. It has a total length of 2400 km and four main branches: the West River, the North River, the East River, and the Liuxi River (Figure 1). The West River and North River merge at the Sixianjiao confluence, and the lower areas are the West River Delta and the North River Delta. The East River Delta is a relatively independent river network that forms the entire Pearl River Delta with the North River Delta. The water and sediment discharge flow through complex river networks and enter the South China Sea through the Shizi Channel and Pearl River Estuary. As a transition area between land and sea, the Pearl River Delta is influenced by fluvial and tidal dynamics, and fluvial dynamics play a dominant role in most regions. The Pearl River hydrologic networks have a great influence on economic development and urban construction.

The water quality and security of the Pearl River are important for the health of the residents living in the surrounding metropolitan areas (e.g., Guangzhou and Dongguan). The combination of ecological values, economic importance, and intensive human interventions makes the Pearl River particularly worthy of concern and study. In the last 40 years, intensive human interventions have strongly disturbed the natural evolution of deltaic channels in the Pearl River. This impact on the material cycle and energy flows in the mainstreams and tributaries of the Pearl River system determines the ecological quality and health. Six river sections (#1 to #6 in Figure 1), extending from the Lixi River confluence to the Pearl River Estuary, were chosen along the main channel of the Pearl River according to land use type and ichthyofaunal distribution (Fan et al. 2019). The habitats in these sections were subjected to long‐term anthropogenic modifications, leading to different habitat types (e.g., agricultural, industrial, island, urban, factory, and estuarine areas) and environmental conditions (e.g., water quality, substrate types, riparian zones, etc.) from #1 to #6, which indicated heterogeneous food web components and structures. Habitat characteristics (Table S1) and physicochemical parameters of water quality (Table S2) were collected according to Method S1 provided in Supporting Information (SI).

### 2.2 Field Sampling and Data Collection

At each sampling site, water, substrate, algae, zooplankton, macroinvertebrate, and fish samples were collected during the rainy (June–July) and dry (November–December) seasons in each year of 2021–2023. In the field, the sampling methods for nonwadable rivers were provided by Wang et al. (2018a). The ratio of annual production to mean biomass (*P*/*B*) of producers and the import of detritus were determined in the field (Method S2 in SI). The *P*/*B* and the ratio of annual consumption to mean biomass (*Q*/*B*) and associated conversion coefficients of fish and invertebrates (Method S3 in SI) were calculated according to the empirical formulae provided by Christensen et al. (2008), Brey (1999), and Kuns and Sprules (2000). The rates of net production and respiration were derived from changes in the concentrations of dissolved oxygen over time in the illuminated and darkened incubations of phytoplankton (Methods S2 and S3 in SI). The stomach/gut samples of the fish and invertebrates were slit, and the contents of these components were analyzed in the laboratory. Gravimetric and volumetric methods (Method S4 in SI) were combined to identify the prey items and quantify their proportions in diet composition (*DC*) matrices. The basic input parameters (e.g., *B*, *P*/*B*, *Q*/*B*, and *UC* [unassimilated consumption]) for the Ecopath models are listed in Table S3 of SI, with the *DC* matrices provided in Table S4 of SI. The biomasses of fish, macroinvertebrates, zooplankton, algae, macrophytes, and detritus were determined in the field from 2021 to 2023, which are assumed to represent the mean values of the ecological data during the past 3 years. The species/genus/family compositions of the fish and invertebrate groups are shown in Table S5 of SI.

### 2.3 Ecopath Modeling

Ecopath modeling was originally designed to provide a simple way to generate information about the standing stock and energy flow of an ecosystem. The basic condition considered for Ecopath analysis is that the energy inputs and outputs of all biomass compartments must be balanced. Two equations are used to parameterize an Ecopath model (Christensen and Walters 2004). The first equation describes the production of each group:

Production = catch + predation mortality + net migration + biomass accumulation + other mortality.

(1) $$\begin{array}{c} {\left(P/B\right)}_{i}\cdot B_{i}=Y_{i}+\sum B_{j}\cdot {\left(Q/B\right)}_{j}\cdot {\mathrm{DC}}_{\mathrm{ij}}+{\mathrm{NM}}_{i}+{\mathrm{BA}}_{i}\\ +{\left(P/B\right)}_{i}\cdot B_{i}\cdot \left(1-{\mathrm{EE}}_{i}\right) \end{array}$$

where for prey *i* and predator *j*, *P*/*B* is the production‐to‐biomass ratio, *B* is the biomass, *Y* is the fishery catch, *Q*/*B* is the consumption‐to‐biomass ratio, *DC*_*ij*_ is the proportion of prey *i* in the diet composition of predator *j*, *NM* is the net migration rate, *BA* is the biomass accumulation rate, *EE* is the ecotrophic efficiency, and (1—*EE*) is the mortality rate other than that from predation and fishing.

The second equation ensures energy balance within each functional group:

Consumption = production + respiration + unassimilated food.

(2) $$Q_{i}=P_{i}+R_{i}+{\mathrm{UC}}_{i}\times Q_{i}$$

where for predator *i*, *Q* is consumption, *P* is production, *R* is respiration, and *UC* is the unassimilated‐to‐consumption rate.

### 2.4 Model Balance Adjustment

Ecological and thermodynamic rules were followed based on a series of logical constraints: 0.1 < gross efficiency < 0.3, gross efficiency < net efficiency, respiration/assimilation < 1, and production/respiration < 1 (Christensen et al. 2008; Odum 1969). To balance the models, it was first evaluated whether the *EE* was < 1 for each group. For groups with *EE* > 1, the biomass, primary production, and fishery yield that were directly measured were left unchanged; the input parameters derived from the literature (e.g., *UC*) or estimated by empirical models (e.g., *P*/*B*, *Q*/*B*) were modified to conform to the constraints. The insufficient supply of prey *i* for predator *j* was adjusted by reducing the proportion of prey *i* in the diet composition of predator *j* (i.e., *DC*_*ij*_) within uncertainty intervals.

The uncertainty of the input data was assessed based on the pedigree index, which was > 0.85 for each model, indicating a high level of credibility. To avoid bias in the estimated values of different parameters, the preliminary data were introduced into Ecoranger, and ranges based on measured relative errors (%) were introduced. Random input variables were drawn from a normal distribution for each basic parameter. The process was repeated to generate a theoretical frequency distribution for each parameter via Monte Carlo simulations. A total of 10,000 models were run, and 200 fitting scenarios were selected to propagate uncertainty from the inputs to the synthetic indices, with the means and standard deviations of the input parameters estimated.

### 2.5 Model Indices and Data Statistics

A series of indices that indicate system maturity (Odum 1969; Ulanowicz 2004), including flow flux, cycling, connectance, organization, and diversity, are calculated in Ecopath (Odum 1969; Ulanowicz 1986). Of special relevance are the following indices: (1) The total system throughput (TST, the sum of all flows within the system), including consumption, respiration, exports, and flows into detritus, provides a measure of ecosystem size; (2) the system omnivory index (SOI) represents an overall measure of the complexity of a given ecological network and thus allows for intercomparison among ecosystems and assessment of their development stage; (3) the connectance index (CI) is correlated with system maturity since the food chain is expected to change from linear to weblike as the system matures; (4) the Finn's cycling index (FCI) is the amount of material or energy flows that are recycled in the food web, and reflects the self‐improvement and health of an ecosystem; (5) the ratio of ascendency to capacity (A/C) measures a system's development in the organization and strength in reserve to unexpected perturbations; (6) the ratio of primary production to respiration (TPP/TR) describes the development of communities, with a value close to 1 indicating maturity; and (7) the ratio of total biomass to TST (TB/TST) is used to assess the biomass supported by available energy, with its value increasing as a system develops from the early phases to mature status.

The transfer efficiency (TE) of the Lindeman spine shows the net amount of flows that each trophic level (TL) receives from the preceding one and the amount that each TL creates for respiration, catches, exports, and flows to detritus. Trophic interactions within the food web were analyzed with the mixed trophic impact (MTI), which is the sum of positive and negative impacts (Christensen and Walters 2004). The MTI was used to assess the trophic impacts of one group by measuring how changes in the biomass of this group affect the biomass of other groups (Ulanowicz and Puccia 1990). Derived from the MTI, Ecopath further calculates a keystoneness (KS) index (Libralato et al. 2006), which is high for functional groups with relatively low biomass but strong MTI; groups with KS values close to or higher than 0 are usually considered keystone.

A correspondence analysis was performed to determine the degree of influence of environmental factors, including the physicochemical parameters of water quality and habitat characteristics, on food web attributes. The relationships between environmental factors and food web attributes were first determined via detrended correspondence analysis (DCA). A DCA1 gradient length > 3.0 (3.56 for water quality and food web attributes and 3.28 for habitat characteristics and food web attributes) indicated a unimodal response; thus, canonical correspondence analysis (CCA) was applied. For efficiency, stepwise forward selection was used to reduce the number of linearly correlated explanatory variables with axes in the CCA. The statistical significance of the axes derived from each analysis was tested with the maximum number of samples via the Monte Carlo test (999 permutations) (Legendre and Legendre 2012). All multivariate analyses were conducted with R.

## 3 Results

### 3.1 Trophic Levels (TLs) and Ecotrophic Efficiencies ( *EE*s) Along the Pearl River

**FIGURE 2:**
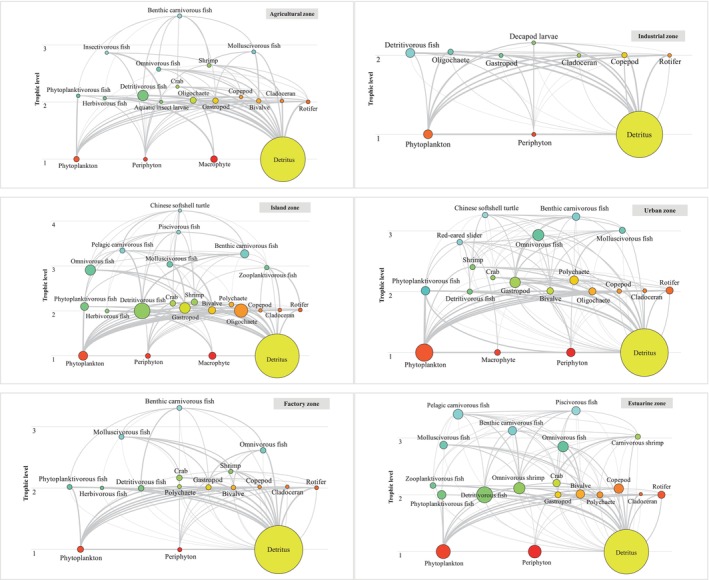
Overview of the mass‐balance flow diagram of the six sampling sections along the Pearl River. The network components are structured along the vertical axis according to their trophic levels. The area of each circle is proportional to the biomass of each group. The width and color of each line are proportional to the diet composition (0%–100%) of each predator group.

Insectivorous fish (0.83, averaged across the 6 sampling sections), aquatic insect larvae (0.96), copepods (0.76), and macrophytes (0.88) presented the highest *EE* values among the fish, macroinvertebrate, zooplankton, and primary producer groups, respectively, indicating that the production of these groups were effectively utilized by consumers and can be transferred to high TLs. In contrast, detritivorous fish, gastropods, annelids, and phytoplankton had the lowest *EE* values, indicating that the production of these groups were less utilized and flowed back to the detritus pool. The six mass‐balance flowcharts (see food web diagrams in Figure 2) revealed that along the Pearl River, softshell turtles (i.e., *Pelodiscus sinensis* in island zone) and piscivorous fish (e.g., *Elopichthys bambusa* and *Piaractus brachypomus* in island zone and *Collichthys lucidus* and *Lateolabrax japonicus* in estuarine zone) were the top predators that occupied the highest TLs. Softshell turtles, which feed on benthic fish and decapods (e.g., shrimp and crabs), presented a TL of 4.41. Piscivorous fish, which feed on pelagic fish and decapods, presented TLs of 3.43–3.48. In agricultural, urban, and factory zones, benthic carnivorous fish, which feed on small‐sized fish and gastropods, were the top predators with TLs of 3.01–3.46. In industrial areas, only detritivorous fish that feed on sedimentary organic detritus were found, and their TL was 2.05. Molluscivorous fish, which feed on gastropods, bivalves, and oligochaetes, presented TLs of 2.46–3.03. Omnivorous fish, feeding on gastropods, bivalves, and phytoplankton, presented TLs of 2.58–2.91. Phytoplanktivorous fish, which feed on phytoplankton, had TLs ranging from 2.00–2.33. The other herbivorous and detritivorous fish that feed on macrophytes or organic detritus had a TL of 2.00.

### 3.2 Biomass and Production

The spatial variation in the biomass and production of fish (*B*_*fish*_ and *P*_*fish*_) revealed that the *B*_*fish*_ and *P*_*fish*_ in estuarine and island zones (24.7–29.7 g/m^2^ and 116–144 g/m^2^/year, respectively) were the highest, whereas those in urban and industrial zones (4.87–11.3 g/m^2^ and 7.94–22.4 g/m^2^/year) were the lowest (Table 1). Similarly, the biomass and production of invertebrates (*B*_*invert*_ and *P*_*invert*_) had the highest values in estuarine and island zones (18.9–22.0 g/m^2^ and 420–696 g/m^2^/year) and the lowest values in urban and industrial zones (3.08–10.7 g/m^2^ and 12.3–275 g/m^2^/year). These results indicate that the biomass and production of consumer functional groups tended to decrease in the following order: estuarine > island > factory > agricultural > urban > industrial zones. Except for the industrial zone, where tolerant oligochaetes (e.g., *Limnodrilus*) accounted for 55.8% of *B*_*invert*_, the composition of *B*_*invert*_ in the other five zones was dominated by gastropods (mean ± SD of 30.3% ± 15.2%), bivalves (17.4% ± 4.03%), shrimp (14.1% ± 7.09%), crabs (11.9% ± 7.36%), rotifers (9.15% ± 3.22%), copepods (6.73% ± 6.15%), and polychaetes (6.59% ± 4.61%). The composition of *P*_*invert*_ in the Pearl River was determined mainly by mollusks (gastropods and bivalves), decapods (shrimp and crabs), zooplankton (rotifers and copepods), and annelids (polychaetes and oligochaetes).

**TABLE 1:** The biomass (g/m^2^) and production flows (g/m^2^/year) of consumers (e.g., fish and invertebrates), producers (e.g., periphyton, phytoplankton, macrophyte), and detritus in six sampling sections along the Pearl River.

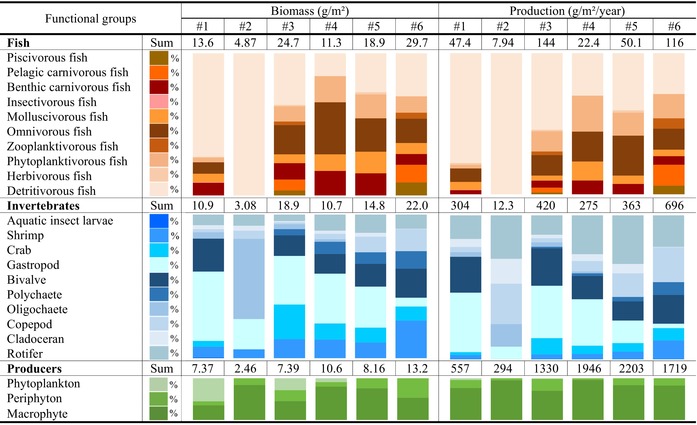

*Note:* The biomass and production flows were decomposed into the percent contribution of each functional group (0%–100% in the stacked bar diagram) through TLs II–V. River sections #1 to #6: Agricultural, industrial, island, urban, factory, and estuarine zones, respectively.

With the exception of the industrial zone, where only exotic detritivorous fish (e.g., Cichliformes) can survive in polluted water, the composition of *B*_*fish*_ in the other five zones was mainly detritivorous (mean ± SD of 36.6% ± 21.5%), omnivorous (21.2% ± 10.4%), phytoplanktivorous (12.3% ± 5.93%), benthic carnivorous (12.1% ± 4.14%), and molluscivorous fish (9.39% ± 3.90%). The pelagic carnivorous, piscivorous, and zooplanktivorous fish, which were only observed in estuarine and island zones, contributed 4.04% ± 5.78%, 2.51% ± 3.97%, and 1.34% ± 1.93%, respectively, to the local *B*_*fish*_. Herbivorous and insectivorous fish, which have high selectivity for habitats (e.g., flowing water and rich hydrophytes), contributed 0.64% ± 0.66% and 0.06% ± 0.14%, respectively, to the local *B*_*fish*_. The patterns of fish functional group contributions to *P*_*fish*_ were similar to those of *B*_*fish*_.

### 3.3 Consumption, Predation, and Unutilized Production

Longitudinally, the consumption of fish and invertebrates (*Q*_*fish*_ and *Q*_*invert*_) was highest in estuarine (696 and 3045 g/m^2^/year) and island zones (528 and 2321 g/m^2^/year) (Table 2). *Q*_*invert*_ was 1.7–10.7 times greater than *Q*_*fish*_, indicating that consumption flows are essentially determined by invertebrates. The composition of *Q*_*fish*_ was mainly detritivorous (mean ± SD of 54.9% ± 30.9%), omnivorous (17.2% ± 13.2%), phytoplanktivorous (12.8% ± 12.2%), molluscivorous (5.05% ± 3.82%), and benthic carnivorous fish (4.67% ± 3.19%). The composition of *Q*_*invert*_ was mainly gastropods (26.1% ± 17.1%), rotifers (25.9% ± 9.27%), bivalves (14.7% ± 8.11%), copepods (12.5% ± 8.70%), and oligochaetes (5.12% ± 8.96%).

**TABLE 2:** The consumption flows (g/m^2^/year) of invertebrate and fish groups through TLs II–V in the six sampling sections along the Pearl River.

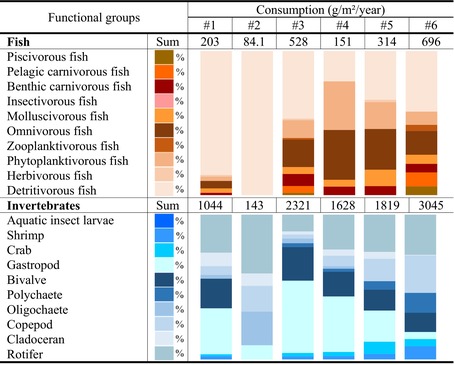

*Note:* The consumption flows are decomposed into the percent contribution of each functional group (0%–100% in the stacked bar diagram) through TLs II –V. River sections #1 to #6: Agricultural, industrial, island, urban, factory, and estuarine zones, respectively.

Detritus contributed the most to predation (53.8% ± 8.09%) and unutilized production (*P*_*unutilized*_) flows (71.5% ± 9.62%), followed by phytoplankton (32.2% ± 8.63% and 17.7% ± 11.9%), invertebrates (7.81% ± 4.55% and 6.26% ± 4.19%), periphyton (5.20% ± 3.39% and 2.74% ± 2.95%), and fish (0.88% ± 0.79% and 1.55% ± 1.21%) (Tables 3 and 4). Predation on invertebrates, which was 4.1–32.9 times greater than that on fish, was determined mainly by rotifers (33.9% ± 19.0%), bivalves (17.9% ± 12.1%), and gastropods (16.7% ± 11.4%). Predation on fish was determined mainly by detritivorous (41.4% ± 7.63%), omnivorous (17.2% ± 8.28%), and molluscivorous fish (12.9% ± 7.32%). The *P*_*unutilized*_ of invertebrates was 1.1–11.2 times greater than that of fish. The patterns of *P*_*unutilized*_ compositions of invertebrates and fish were similar to those of predation on invertebrates and fish.

**TABLE 3:** Predation flows (consumption on prey = predatory mortality of prey × production of prey, g/m^2^/year) through TLs II–V in the six sampling sections along the Pearl River.

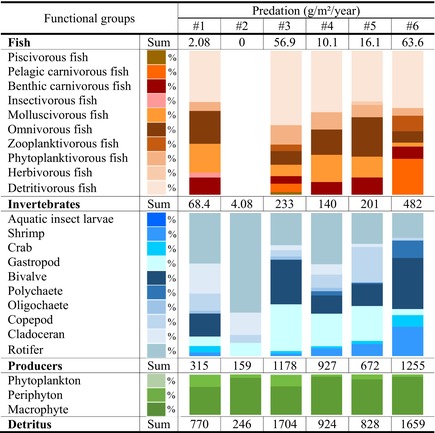

*Note:* The predation flows are decomposed into the percent contribution of each functional group (0%–100% in the stacked bar diagram) through TLs II–V. River sections #1 to #6: Agricultural, industrial, island, urban, factory, and estuarine zones, respectively.

**TABLE 4:** Unutilized production (*P*_unutilized_ = [1 − *EE*] × production, g/m^2^/year) flowing back to detritus in the six sampling sections along the Pearl River.

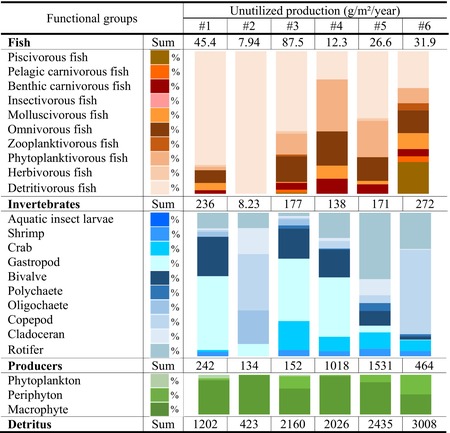

*Note:* The unutilized production flows were decomposed into the percent contribution of each functional group (0%–100% in the stacked bar diagram) through TLs II–V. River sections #1 to #6: Agricultural, industrial, island, urban, factory, and estuarine zones, respectively.

### 3.4 Transfer Efficiencies (TEs) of the Lindeman Spine

**FIGURE 3:**
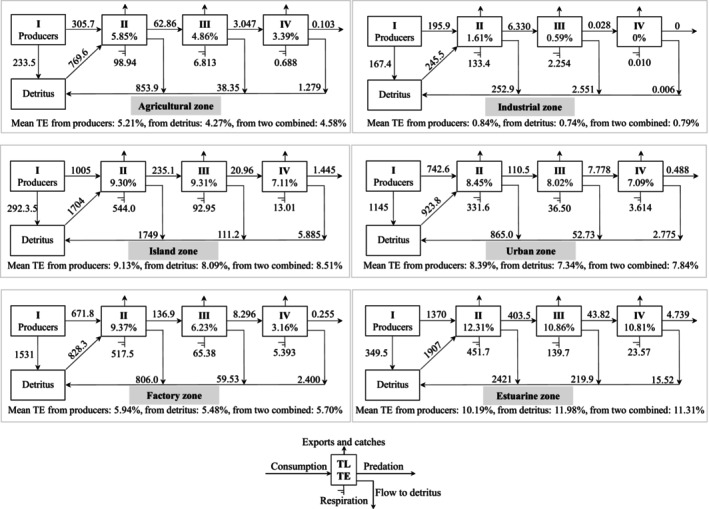
Simplified diagrams of the energy transfers originating from producers and detritus between discrete trophic levels (TLs, I–IV) in the six sampling sections along the Pearl River. The percentage (%) values indicate the trophic efficiency (TE) per TL calculated by combining producers and detritus. The separated partial flows consisted of the consumption of the last TL, the predation by the next TL, exports and catches from the system, respiration, and flow to detritus. The numbers and arrows indicate the flow of energy expressed in g/m^2^/year.

The mean TE values calculated from producers, detritus, and the sum of producers and detritus in estuarine (10.2%, 12.0%, and 11.3%) and island zones (9.13%, 8.09%, and 8.51%) were the highest, whereas those in agricultural (5.21%, 4.27%, and 4.58%) and industrial zones (0.84%, 0.74%, and 0.79%) were the lowest (Figure 3). The decomposed TEs at each TL showed that the highest TE values at TLs II–IV were recorded in estuarine (12.3%, 10.9%, and 10.8%) and island zones (9.30%, 9.31%, and 7.11%). The lowest TE values at TLs II–IV were recorded in the industrial zone (1.61%, 0.59%, and 0%). Generally, the highest TE values were found in estuarine and island zones, where the predation flows from TLs II to III (403.5 and 235.1 g/m^2^/year) and from TLs III to IV (43.8 and 21.0 g/m^2^/year) were also the highest. This indicates that the high TE values of the whole food web depended on the predation flows from the lower TL to the higher TL; in particular, the TEs of TLs II and III were the main factors that determined the TE of the entire food web. The consumption flows along the food chain revealed that pelagic planktivorous fish filtering on zooplankton and molluscivorous or omnivorous fish feeding on bivalves or softshell gastropods are two important functional groups that determine the TE of the food web. Notably, the TE value of TL IV in the industrial zone was 0% due to the lack of predators at high TLs (e.g., carnivorous fish), resulting in the limitation of energy transmission from TL III to higher TLs.

### 3.5 Keystoneness (KS) and Mixed Trophic Impacts (MTIs)

**FIGURE 4:**
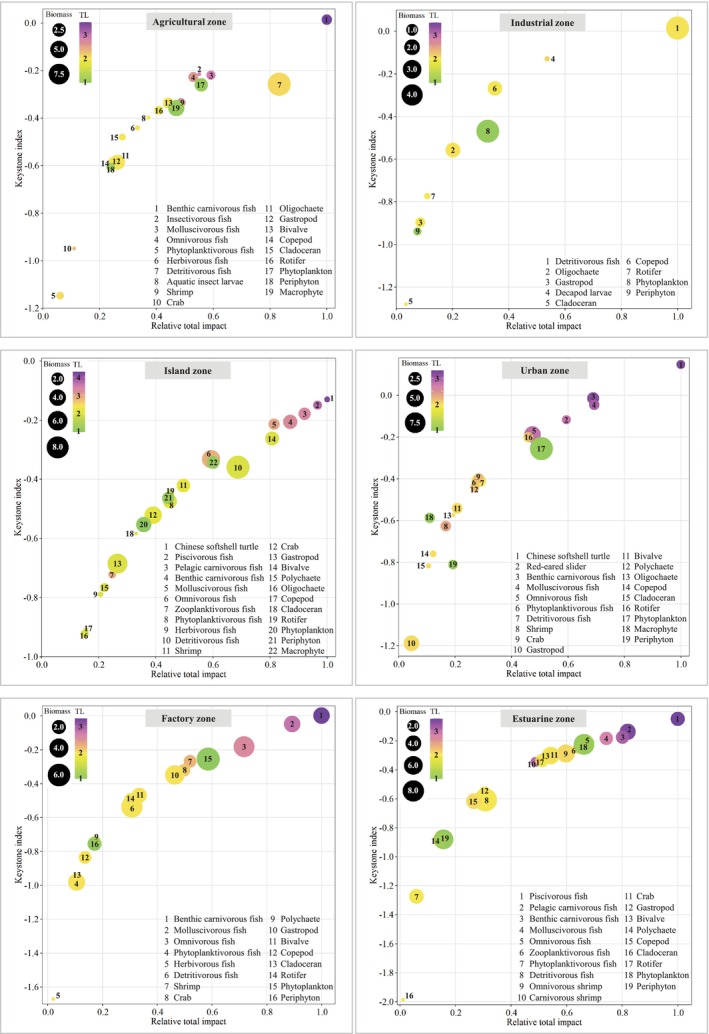
Keystone indices for the functional groups of the six trophic webs along the Pearl River. For each group, the keystone index (*y*‐axis) is reported against the relative total impact (*x*‐axis). The total impact is relative to the maximum impact measured in each trophic web; thus, for the *x*‐axis, the scale is always between 0 and 1. The area and color of each circle are proportional to the biomass and trophic level (TL), respectively, of each group.

**FIGURE 5:**
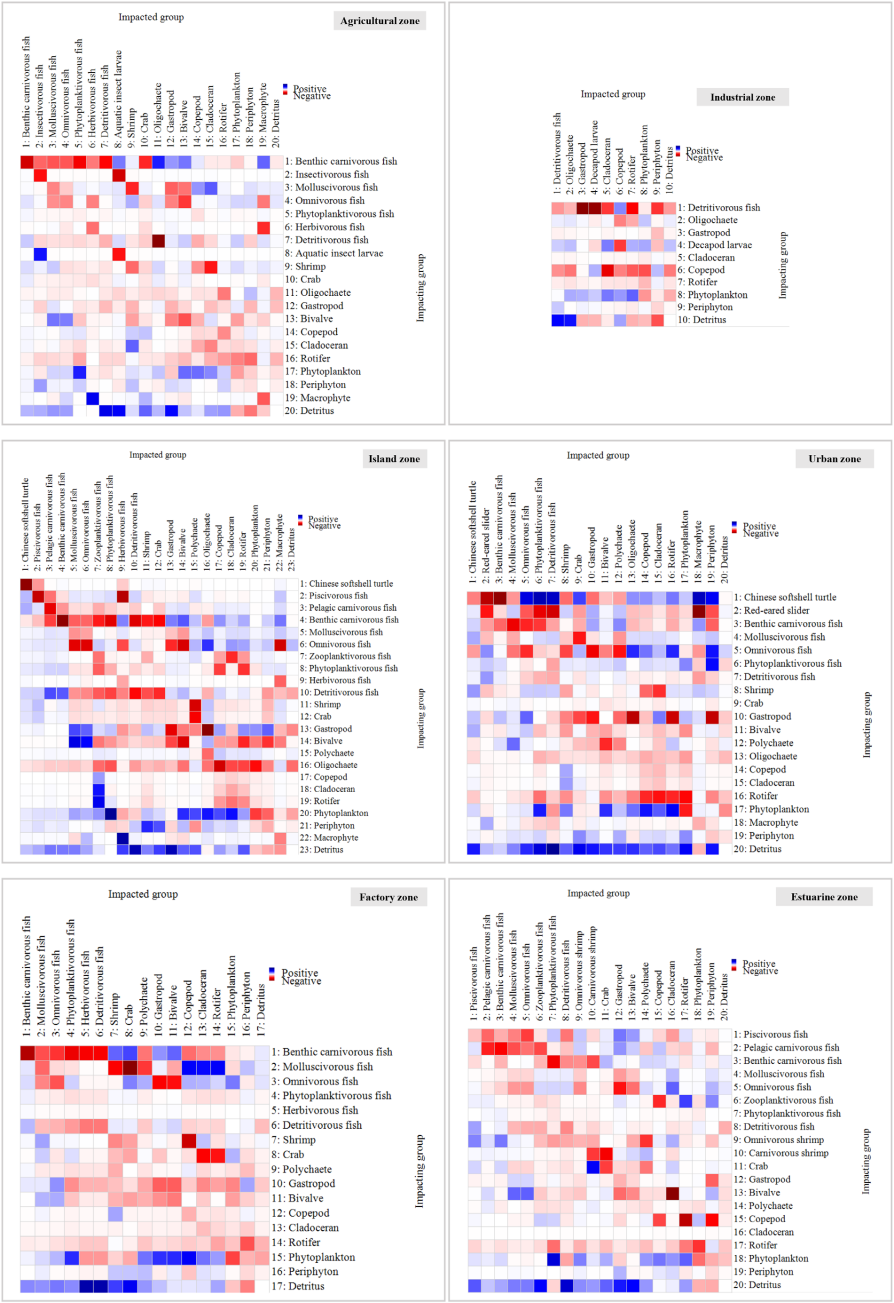
Mixed trophic impacts of the six Ecopath models along the Pearl River. The magnitude of the impact is represented by the darkness of blue (positive impacts) and red (negative impacts). The impacting and impacted groups are listed to the left of (row headings) and above (column headings) the histograms, respectively.

The functional groups with the highest KS (Figure 4) were benthic carnivorous fish (0.001–0.014) and molluscivorous fish (−0.214 to −0.048) in agricultural and factory zones; detritivorous fish (0.015) and decapod larvae (−0.129) in the industrial zone; softshell turtles (−0.127), piscivorous fish (−0.149), and pelagic/benthic carnivorous fish (−0.206 to −0.178) in the island zone; softshell turtles (0.148) and benthic carnivorous fish (−0.014) in the urban zone; and piscivorous fish (−0.049) and pelagic/benthic carnivorous fish (−0.176 to −0.139) in the estuarine zone. Except for primary producers and detritus, which had great positive impacts on all the consumers, the functional groups with the strongest MTIs (Figure 5) were benthic carnivorous fish (4.38), detritivorous fish (2.06), and molluscivorous fish (1.92) in the agricultural zone; detritivorous fish (2.58) and decapod larvae (2.25) in the industrial zone; benthic carnivorous fish (3.37), omnivorous fish (2.62), and gastropods (2.25) in the island zone; softshell turtles (5.27) and benthic carnivorous fish (3.43) in the urban zone; benthic carnivorous fish (3.92) and molluscivorous fish (3.07) in the factory zone; and pelagic/benthic carnivorous fish (3.80–3.88), piscivorous fish (3.16), and bivalves (3.01) in the estuarine zone. Both the KS and MTI results indicated that predators at the apex of the food web exerting “top‐down” control was the basic trophic pattern along the Pearl River. Mollusks as food resources exerted “wasp‐waist” control in island and estuarine zones, and detritivorous fish and decapod larvae, which exerted “bottom‐up” control in the industrial zone, exhibited specific trophic patterns.

### 3.6 Ecosystem Theory and Information Indices of the Pearl River

The ecosystem theory and information indices (Table 5) revealed that the six river systems could be classified into four levels. The first‐level systems were represented by estuarine and island zones, which presented the highest values of TST (5396–6890 g/m^2^/year), FCI (32.8–35.0), Finn's mean path length (FML, 8.50–9.00), SOI (0.098–0.133), Shannon diversity index (2.555–2.620), and CI (0.313–0.370); meanwhile, the TPP/TR in island (2.04) and estuarine zones (2.78) were also close to 1. The pattern of these attribute values indicated that the system status (e.g., stability, maturity, and diversity) of the estuarine and island zones was healthier than that of the other zones. The second‐level systems were represented by the urban (#4) and factory (#5) zones, which had the second‐highest values of TST (4495–5459 g/m^2^/year), FCI (20.1–31.1), FML (5.61–7.42), SOI (0.065–0.073), Shannon diversity index (2.311–2.527), and CI (0.257–0.259). The third‐level system was represented by the agricultural (#1) zone, which presented low values of TST (2462 g/m^2^/year), FCI (15.1), FML (5.31), SOI (0.050), Shannon diversity index (2.217), and CI (0.248). The fourth‐level system was represented by the industrial (#2) zone, which presented the lowest values of TST (677 g/m^2^/year), FCI (13.5), FML (3.55), SOI (0.024), Shannon diversity index (1.571), and CI (0.235), indicating that the local food web structure had been severely damaged and the system was unhealthy.

**TABLE 5:** Summary statistics and network flow indices of the six Ecopath models along the Pearl River.

Parameter	Unit	Agricultural zone	Industrial zone	Island zone	Urban zone	Factory zone	Estuarine zone
Ecosystem theory indices							
Sum of all consumption	g/m^2^/year	1247	227	2850	1780	2133	3741
Sum of all respiration (TR)	g/m^2^/year	306	136	651	472	710	618
Sum of all back flows to detritus	g/m^2^/year	1127	423	2160	2066	2399	3006
Total system throughput (TST)	g/m^2^/year	2462	677	5396	4495	5459	6890
Sum of all production	g/m^2^/year	909	314	1895	2243	2616	2531
Total net primary production (TPP)	g/m^2^/year	557	294	1330	1946	2203	1719
Net system production (TPP‐TR)	g/m^2^/year	250	158	679	1474	1493	1101
Total biomass (TB, excluding detritus)	g/m^2^	31.9	10.4	51.3	34.4	41.8	65.6
Total primary production/total respiration (TPP/TR)		1.82	2.16	2.04	4.12	3.10	2.78
Total primary production/total biomass (TPP/TB)		17.5	28.2	25.9	56.6	52.6	26.2
Total biomass/Total system throughput (TB/TST)		0.0129	0.0154	0.0095	0.0076	0.0077	0.0096
Cycle, connectance, and diversity indices							
Throughput cycled (excluding detritus)	g/m^2^/year	2.49	0	7.03	6.12	4.67	8.54
Throughput cycled (including detritus)	g/m^2^/year	683	280	1878	878	897	2199
Predator cycling index	%	0.235	0	0.541	0.506	0.451	0.740
Finn's cycling index (FCI)	%	15.1	13.5	35.0	31.3	20.1	32.8
Finn's mean path length (FML)		5.31	3.55	8.50	7.42	5.61	9.00
Finn's straight‐through path length (excluding detritus)		2.58	2.05	4.34	3.29	2.83	3.37
Finn's straight‐through path length (including detritus)		3.65	3.02	6.05	4.85	4.72	6.79
Connectance Index (CI)		0.248	0.235	0.313	0.259	0.257	0.370
System Omnivory Index (SOI)		0.050	0.024	0.098	0.073	0.065	0.133
Shannon diversity index		2.217	1.571	2.555	2.527	2.311	2.620
Information indices							
Capacity (C)	Flowbits	13,368	6483	27,016	19,338	23,967	34,867
Ascendency (A)	Flowbits	2248	1560	5429	3384	4393	6946
Ascendency/Capacity (A/C)	%	16.8	24.1	20.1	17.5	18.3	19.9

### 3.7 Influence of Environmental Factors on Food Web Attributes

**FIGURE 6:**
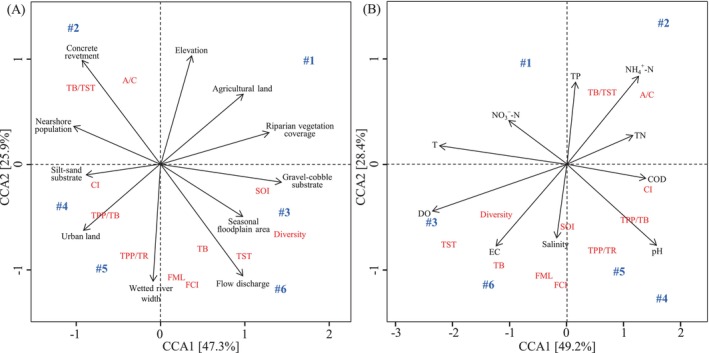
Canonical correspondence analysis (CCA) between environmental factors and food web attributes. (A) shows the CCA between habitat characteristics (see details in Table S1) and food web attributes. (B) shows the CCA between the physicochemical parameters of water quality (see details in Table S2) and food web attributes. River sections #1 to #6: Agricultural, industrial, island, urban, factory, and estuarine zones, respectively. Habitat characteristics: Wet stream width (m), Flow discharge (m^3^/s); Seasonal floodplain area (m^2^); Gravel‐cobble substrate (% of substrate area); Silt‐sand substrate (% of substrate area); Urban land (% of land area); Agricultural land (% of land area); Riparian vegetation coverage (%); Nearshore concrete revetment (km); and Nearshore population (thousand). Water quality parameters: T, water temperature (°C); DO, dissolved oxygen (mg/L); EC, electrical conductivity (μs/cm); COD, chemical oxygen demand (mg/L); TN, total nitrogen (mg/L); NH_4_^+^‐N, ammonia nitrogen (mg/L); NO_3_^−^‐N, nitrate nitrogen (mg/L); TP, total phosphorus (mg/L), Salinity (‰). Food web attributes: Diversity, Shannon diversity index; CI, connectance index; TST, total system throughput (g/m^2^/year); TB, total biomass (g/m^2^); TB/TST, the ratio of TB to TST; TPP/TR, the ratio of total primary production to total respiration; TPP/TB, the ratio of total primary production to TB; FCI, Finn's cycling index; FML, Finn's mean path length; A/C, the ratio of ascendency to capacity; SOI, system omnivory index.

Canonical correspondence analysis (CCA) between the environmental factors and food web attributes revealed that the six sampling zones could be divided into four groups distributed in four quadrants (Figure 6). In the second quadrant of Figure 6A and the first quadrant of Figure 6B, the food web attributes in industrial zone (#2) with poor water quality (e.g., high concentrations of nitrogen and phosphorus and low DO) and degraded riparian habitats (e.g., large areas of urban land use and long concrete revetment distances) were immature (high TPP/TR and low throughput cycles), unstable (e.g., low CI and SOI), and simplified (e.g., short food chain length). In contrast, in the fourth quadrant of Figure 6A and third quadrant of Figure 6B, the food web attributes in island (#3) and estuarine (#6) zones, with less pollution and diverse habitats, exhibited mature (e.g., TPP/TR close to 1 and high throughput cycled), stable (e.g., high CI and SOI), and complex (e.g., long food chain length) system statuses. River section #2 was a typical zone that indicated serious anthropogenic interference caused by industrial development (e.g., factory construction and water pollution); as a result, local food web attributes tended toward underdevelopment and immaturity. Island zone #3 was the only pristine habitat with less human disturbance, where both the water quality and habitat heterogeneity were high. Although estuarine zone #6 also experienced water pollution and habitat degradation, the local functional groups were the richest because both freshwater and brackish fish and invertebrates converged there; thus, a series of food web attributes indicating ecosystem maturity and stability were high. The relationships between the environmental factors and food web attributes in agricultural (#1), urban (#4), and factory (#5) zones indicated moderate disturbances, which were greater than those of #3 but less than those of #2.

## 4 Discussion

The spatial variation in food web attributes was closely associated with changes in environmental factors and the composition of functional groups along the Pearl River. The appearances of softshell turtles, piscivorous fish, and herbivorous/molluscivorous fish can be used to indicate the integrity of local food web structures. The increase in exotic species and the decrease in native top predators are two factors resulting in the low efficiency of energy transmission. The food web structure in the industrial zone collapsed due to water pollution and habitat degradation. In contrast, the undisturbed island zone, which provides essential feeding/spawning habitats for fish, could be protected as a multifunctional food web area to maintain river health.

### 4.1 Regional Patterns of the Highest TLs and TEs Along the Pearl River

The highest TL in the Pearl River was 4.41 for softshell turtles, followed by piscivorous fish at 3.43–3.48, which was similar to the TL reported in freshwater systems (Wang et al. 2021a) and much lower than that reported in marine systems (commonly > 4.5) (Keramidas et al. 2023). The softshell turtle ( *P. sinensis* ) and piscivorous fish ( *E. bambusa* , *P. brachypomus* , *C. lucidus* , and *L. japonicus* ) were the top predators with the highest TLs in the Pearl River; however, their populations are constantly declining due to increased anthropogenic interference, such as water pollution, habitat degradation, and fishing pressure (Wang et al. 2020b). The direct impacts of decreasing top predator biomass were decreased energy flows from low TLs to high TLs, which led to low Lindeman TEs and weakened MTIs (Du et al. 2020; Wang et al. 2021a).

The highest TEs recorded in island and estuarine zones could be attributed to the appearance of softshell turtles (e.g., *P. sinensis* ) and piscivorous fish (e.g., native *E. bambusa* and invasive *P. brachypomus* ) as well as the molluscivorous fish (e.g., *Mylopharyngodon piceus* ) at TLs III–IV. These high‐level groups facilitated energy transmission from low TLs to high TLs (Wang et al. 2018a); for example, the consumption of detritivorous fish by carnivorous fish and the consumption of bivalves and gastropods by molluscivorous fish increased the predation flows from TL II to TL III (Wang et al. 2021b). In addition, compared with freshwater zones, more planktivorous fish feed on zooplankton (e.g., copepods) in estuarine brackish waters, which is an important pathway for upward energy transmission. In contrast, the extremely low TEs in the industrial zone were caused by the disappearance of most fish functional groups, with the exception of detritivorous fish (e.g., tolerant tilapia), and thus, large amounts of energy were retained at TL II.

### 4.2 Keystone Groups and Their MTIs on River Food Webs

The keystone groups in the Pearl River are composed of top predators (e.g., softshell turtles, pelagic piscivorous fish, and benthic carnivorous fish at TLs III–IV) and medium predators (e.g., molluscivorous and omnivorous fish at TLs II–III), indicating that the MTIs of Pearl River food webs were determined mainly by top‐down control (Heymans et al. 2016). The high keystoneness of top predators could be attributed to their strong negative impacts (e.g., predation stress) on prey groups and their low biomass due to multiyear sexual maturity and slow growth (Binion‐Rock et al. 2023). Thus, small changes in the biomass of top predators can lead to large MTIs on other functional groups. In the industrial zone, detritivorous fish and decapod larvae were the keystone groups, indicating that bottom‐up control was the main trophic effect in the degraded food web (Fortuna et al. 2024). The observed patterns in the Pearl River were consistent with the theoretical findings of Christen (2004), who suggested that bottom‐up control was commonly found in the initial ecosystem, with large amounts of redundant primary production or a degraded food web experiencing water pollution (Bentley et al. 2024).

Interestingly, although top predators such as softshell turtles and piscivorous fish had negative impacts on other fish groups, they had positive impacts on invertebrate groups (through decreasing the competition between invertebrates) and primary producers (through decreasing the predation pressure on primary producers) (Wang et al. 2018a). The predation by top predators can improve energy transmission from low TLs to high TLs and simultaneously control overproduction by primary producers (Wang et al. 2019b), which are beneficial to enhance system attributes (e.g., increase in omnivory, connectance, and diversity). However, the biomass of top predators has decreased sharply in recent years because of the deteriorating environment (e.g., water pollution and habitat degradation) and fishing pressure due to their high market value (Wang et al. 2023b). Therefore, we can conclude that the conservation of top predator populations and the restoration of their essential habitats are important for maintaining the integrity and health of river ecosystems.

### 4.3 Ecosystem Attributes and Their Indications of Ecological Evaluation

A series of indices that characterize the maturity of a system (Odum 1969; Ulanowicz 2004), including flows (e.g., low TPP/TB, high TB/TST, TPP/TR close to 1), cycles (e.g., high FCI and FML), connectance (e.g., high CI), and diversity (e.g., high Shannon index), revealed that the island zone in the middle river and the estuarine zone near the river mouth were more mature and healthier than other zones (Christensen 1995; Heymans et al. 2016). With less human disturbance, the isolated island zone has a pristine landform and provides essential habitats (e.g., emergent and submerged vegetation) for diverse communities (e.g., reptiles, herbivores, and molluscivores). Similarly, the estuarine zone provides a transition zone for both freshwater and brackish habitats, and both fluvial and marine communities could be found in this zone, where the food web structure was diverse and closely connected (i.e., high SOI and CI) (Geers et al. 2016). As a result, the direct advantages of heterogeneous habitats on the island and estuarine habitats were suitable for energy flow along specific food chains, such as “detritus—decapods—carnivores—piscivores”, “macrophytes—herbivores—carnivores”, and “POM/phytoplankton—zooplankton—planktivores” (Morales‐Zárate et al. 2011). These food chains facilitate the energy flows from low TLs to high TLs, which promotes the systemic capacity to carry more energy flows (e.g., total consumption and system throughput) with less redundancy. As suggested by Wang et al. (2024a), heterogeneous habitats can enhance the MTIs among predator and prey groups; thus, the submerged vegetation zone surrounding the island zone is important for promoting the development of the ecosystem toward maturity. In contrast, the low maturity of the agricultural zone was caused by eutrophication and excessive production of phytoplankton, which led to a large amount of unutilized production (i.e., high *P*_*unutilized*_ value). The low maturity of urban and factory zones was caused by the lack of specific (e.g., herbivorous and molluscivorous) food chains, which led to system degradation to the ‘early developmental stage’ (Odum 1969). In particular, the TPP/TR in industrial zone were far less than 1, indicating that the local system was under great pressure from extraneous interference, i.e., organic pollution in industrial zone.

### 4.4 Disturbance Factors That Lead to the Degeneration of the Pearl River Food Web

Through the output of the Ecopath model, we determined that water pollution, overfishing, and exotic invasion risk were three key factors that led to the degeneration of the river food web. A destroyed ecosystem was observed in the industrial zone (#2), where there were no piscivorous or carnivorous fish due to heavy contamination (e.g., discharge of domestic and industrial sewage into the river). In this zone, invasive fish and invertebrates, such as *Coptodon zillii*, *Cirrhinus mrigala* , *Pterygoplichthys pardalis* , and *Pomacea canaliculata* , consume large amounts of organic detritus in sediment, and the local TLs are as low as 2.0 (Wang et al. 2018b). Although top predators could still be sampled in agricultural and urban zones, their biomasses have decreased continuously during recent decades due to overfishing by local fishermen. For example, migratory *Tenualosa reevesii* , which feeds on copepods and diatoms, has disappeared in the Pearl River because of water pollution and overfishing. Other benthic fish, such as *Pelteobagrus fulvidraco* and *Silurus asotus*, sharply decreased in biomass. In contrast, owing to the favorable environment (e.g., no seasonal drought occurred in Africa) and the lack of natural enemies (e.g., South American *Arapaima gigas* , which can eat *P. pardalis* ), invasive species propagate rapidly and form high biomass, leading to the redundancy of energy flows remaining at a TL of 2.0. Exotic species from Africa, South America, and Thailand have been reported to influence ecological niches (e.g., spawning, nursing, and feeding); however, our results further demonstrated that exotic species increased unutilized production and decreased Lindeman TE, which led to the immaturity and instability of the food web. For example, the redundant production of *P. canaliculata* and *Oreochromis niloticus* cannot be utilized because there are no natural enemies in the Pearl River.

### 4.5 Indicator Groups/Species, Food Chains, and Pristine Habitats That Should Be Given Sufficient Attention and Protection

Most researchers and government agencies are concerned with the recruitment and release of native species; however, there is little evidence about how to protect and restore river ecosystems at the food web level. In the Pearl River, top predators are keystone groups that exhibit “top‐down” control, and their MTIs are important for maintaining system balance. In particular, softshell turtles with their juveniles can be found at the outlet of the urban channel connected with the Pearl River; this shoal area is important for turtle spawning and feeding and should receive attention and protection. In the island zone, the graze‐feeding food chain accounts for a large proportion of the energy flow from macrophytes to herbivorous fish, which could be explained by the high biomass of *Ctenopharyngodon idella* —an important indicator species of habitat degradation (Wang et al. 2018b). Because of the increasing urban land use in riparian zones during the past few decades, pristine riparian vegetation has been replaced by concrete revetments, which have had great negative impacts on the feeding and nurseries of herbivorous fish, leading to a notable decline in herbivorous populations in the Pearl River. Therefore, we suggest protecting shoal and island areas where sand beaches and aquatic vegetation are still present from being buried or made artificial, which is important for sustaining ecosystem health.

An interesting finding was that the redundant energy flows retained in TL II were caused by unutilized mollusk production, which could be attributed to the continuous decrease in molluscivorous fish biomass during the past few decades. For example, the disappearance of *M. piceus* was a major reason for the decreased predation on mollusks (e.g., *Cipangopaludina* and *Bellamya*), leading to decreased energy flows from TL II to TL III. Notably, most top and middle fish predators in the disturbed Pearl River face the same problems: (1) migratory species (e.g., *M. piceus* and *Anguilla japonica* ) cannot swim back to spawning grounds due to water conservancy projects; (2) wild populations are decreasing due to overfishing and water pollution; and (3) population density is difficult to restore due to slow growth and a long sexual maturity cycle (Wang et al. 2021a). In conclusion, the unhealthy status of the Pearl River food web was caused mainly by the decline or break of predator–prey links with high energy transfer pathways, such as molluscivorous and herbivorous food chains (Wang et al. 2018a). Our results provide important evidence for ecosystem protection from the aspects of keystone groups, food chains, and habitat areas.

### 4.6 Best Practices of the Ecopath Model for the Evaluation and Management of River Health

After multiple case studies, we believed that the parameters that could reflect the core food web attributes and ecosystem status were biomass, predation, and *P*_*unutilized*_. Unlike biomass, which is obtained via actual measurements in the field, other input parameters, such as *P*/*B*, *Q*/*B*, and *EE*, are commonly obtained via empirical algorithms. Thus, field‐obtained biomass reflects the dominant classification and relative importance of food web components (e.g., functional groups). Predation is more important than production and consumption because predation can provide comprehensive feedback on TEs and energy flow amounts along predator–prey links, which influence Finn's path length, cycling, and connectance. *P*_*unutilized*_ is a crucial parameter that determines the parameters associated with TPP/TR and TB/TST. Some classical restoration cases use top predators to control zooplanktivorous fish and filter‐feeding fish to control algal blooms. From the perspective of energy flow, these ecological engineering methods could be explained by MTIs among functional groups, increasing energy flows from low TLs to high TLs and Lindeman TEs between TLs. With the help of the Ecopath model, eco‐environmental managers could stimulate how much biomass should be added to the ecosystem through enhancement and release, which can help managers to develop scientific policies through a quantitative method. Thus, we suggest that the Ecopath model can identify more ecological problems than a traditional survey of the population or community can do; meanwhile, eco‐environmental managers can obtain more information on how to restore the ecosystem and make more scientific policies, such as controlling algae and stock enhancement—the release of cultured juveniles into the wild population.

## 5 Conclusion

The highest TL in the Pearl River was 4.41 for softshell turtles, followed by 3.43–3.48 for piscivorous fish. The highest TEs recorded in island and estuarine zones could be attributed to the appearance of softshell turtles and piscivorous fish as well as the molluscivorous fish at TLs III–IV. The spatial variation in the food web attributes of the Pearl River showed that the island and estuarine zones were more mature than the other zones were. An undisturbed island zone with submerged vegetation, which provides essential feeding and spawning habitats, should be protected to maintain river health. The food web in the industrial zone collapsed due to water pollution and habitat degradation. The appearance of softshell turtles, black carp, and grass carp can be used to indicate the integrity of the food web structure. The increase in exotic species and the decrease in native top predators are two factors that result in the low efficiency of energy transmission. Future river management should pay critical attention to the health and functioning of aquatic food webs.

## Author Contributions

**Sai Wang:** conceptualization (lead), data curation (lead), formal analysis (lead), funding acquisition (equal), investigation (equal), methodology (equal), project administration (equal), writing – original draft (lead), writing – review and editing (lead). **Yong‐Duo Song:** data curation (equal), formal analysis (equal), investigation (equal). **En‐Ni Wu:** data curation (equal), formal analysis (equal), investigation (equal), methodology (equal), writing – original draft (equal). **Tuan‐Tuan Wang:** conceptualization (lead), data curation (equal), formal analysis (equal), funding acquisition (equal), investigation (equal), methodology (equal), project administration (equal), writing – original draft (lead), writing – review and editing (lead). **Muhammad Shahid Iqbal:** data curation (equal), formal analysis (equal). **Hui‐Long Ou:** data curation (equal), formal analysis (equal), investigation (equal), methodology (equal). **Jia Xie:** data curation (equal), formal analysis (equal), investigation (equal), methodology (equal). **Wen‐Tong Xia:** data curation (equal), formal analysis (equal), investigation (equal), methodology (equal). **Feng‐Juan Yang:** data curation (equal), formal analysis (equal), investigation (equal), methodology (equal). **Jie Feng:** data curation (equal), formal analysis (equal), investigation (equal), methodology (equal). **Shen‐Hao Wu:** data curation (equal), formal analysis (equal), investigation (equal), methodology (equal). **Yang Zhang:** data curation (equal), formal analysis (equal), investigation (equal). **Cong‐Cong Jin:** data curation (equal), formal analysis (equal), investigation (equal). **Zhuo‐Luo Ma:** investigation (equal), methodology (equal). **Hong‐Jin Zhang:** data curation (equal), formal analysis (equal), investigation (equal). **Li‐Yong Miao:** investigation (equal), methodology (equal). **Kuan‐Song Wang:** investigation (equal), methodology (equal).

## Conflicts of Interest

The authors declare no conflicts of interest.

## Supporting information


Data S1.

## Data Availability

The data that supports the findings of this study are available in the Supporting Information of this article.
