# Supplementary material for: Spatial Variation in Food Web Structures, Energy Flows, and System Attributes Along the Pearl River and Their Indications of Protection and Restoration
Source: Ecol Evol. 2025 Feb 24;15(2):e70991. doi: 10.1002/ece3.70991 (PMC11850446; doi:10.1002/ece3.70991)
Supplement: Supplementary file 1 — Data S1. [file ECE3-15-e70991-s001.docx]

**Supporting Information**

**Appendix A Supplemental Methods**

**Method S1 Monitoring methods for physicochemical parameters of water quality**

At each river section, channel width (m), depth (m), and current velocity (m·s^−1^) were measured using a GPS, portable [propeller](app:ds:propeller) flow meter (LS25-3C, Tongda, China), and depth finder (SM-5A, Speedtech, USA), respectively. Temperature (°C), dissolved oxygen (mg·L^-1^), pH, and electrical conductivity (μS·cm^−1^) were measured using a YSI meter (YSI Pro Plus, Yellow Springs, OH, USA). To determine total suspended matter (TSM) and chlorophyll *a* concentration (Chl-*a*), 2 L and 500 mL of water were filtered through pre-combusted (at 450 °C for 6 h) glass fiber filters (Whatman GF/F, *n* = 6) and cellulose acetate membrane filters (0.45 μm, *n* = 6), respectively. Samples for total nitrogen (TN), nitrate nitrogen (NO_3_^‒^-N), [ammonia](app:ds:ammonia) [nitrogen](app:ds:nitrogen) (NH_4_^+^-N), and total [phosphorus](app:ds:phosphorus) (TP) were preserved in acid-washed 300 mL polyethylene bottles (for each *n* = 6). All filters and water samples collected in the field were stored at -18 °C. In the laboratory, TSM filters were dried at 105 °C to constant weight; for pre-treatment steps, Chl-*a* was extracted from homogenized filters using acetone, NO_3_^‒^-N and NH_4_^+^-N samples were filtered through Whatman GF/F, and TN and TP samples were digested with alkaline persulfate at 120 °C. Leaching solutions of Chl-*a*, digested TN and TP, and filtered NO_3_^‒^-N and NH_4_^+^-N were determined using colorimetric methods. All water quality parameters were determined according to standard methods for water and wastewater analysis (SEPA 2002).

**Method S2 Sampling methods of different functional groups in nonwadeable sections of the Pearl River**

| Items | Sampling methods |
| --- | --- |
| Nekton (fish, shrimp, and crab) | Electrofishing equipment (Huayi, China) was used with a 24-kW generator, a 12V-160A lithium battery, a silicon-controlled inverter and a voltage-frequency regulator. A 1-4 m-long telescopic copper cathode was used to efficiently conduct sampling in a water column that was 1.5 m wide × 1.5 m long × 2.5 m deep. A 20-cm diameter ring anode with a 3-mm mesh net was used to capture stunned nekton. In non-wadeable river, the equipment was used at high voltage with main frequency. A 8 m-long welded hull boats was used for boat-electrofishing in deep waters, which was propelled by bamboo quant to reduce noise at a movement speed 3 m min^-1^. The distance covered by the boat was 500 m, spanning both river-banks across various habitats. |
| Macroinvertebrates | 1) > 1 m depth, 1/16 m^2^ Petersen grab bucket sampler was used to evenly sample across the main channels;  2) < 1 m depth, 0.3 × 0.3 m^2^ D-frame net was used in near-shore areas, each sweep in 0.5-m length. |
| Zooplankton | 1) Depth-integrated composite samples of phytoplankton and zooplankton are collected;  2) In the deepest portion of each site, from 0.5 m above the bottom to the surface, with a plankton net (160 μm nylon mesh) |
| Phytoplankton | 1) In the nutrient-enriched middle-lower river, depth-integrated water samples were quantified in 1-L volume and filtered through glass fibers; Chl-*a* were extracted using 90 % acetone, stored for 24 hours at 4 ^o^C in the dark, and determined by spectrophotometric technique;  2) Net production (NP) and community respiration (CR) were derived from the change in DO concentration in replicate light and dark 300 ml borosilicate bottles during 24 h incubation (Bott et al. 1978, Hauer and Lamberti 2007). |
| Periphyton | 1) Periphyton attached to the substratum, measuring 2 cm wide × 2cm long, was gently scraped from the substrate (e.g., cobble, gravel, or brick surface). Areal periphyton biomass for each subregion was calculated by multiplying the biomass per square meter of substrate by the total substrate area available in each subregion, and then dividing that by the surface area of each subregion;  1) Production rates of periphyton were determined by the changes in dissolved oxygen (DO) concentrations in microcosm incubations using water column only and periphyton+water column. Each microcosm consisted of a 3.75-L (10 cm wide × 25 cm long ×15 cm deep) transparent chamber with an opening for inserting probe at the top.  2) Dark microcosms with 100% shading were tightly wrapped in aluminum foil for complete exclusion of irradiance to determine respiration rates;  3) The substates that covered by periphyton were put into the microcosm for 24 hours, and the DO concentrations were determined each 2 hours by a DO instrument (YSI 5100, USA) with a propeller to dissolve vertical gradients in oxygen concentration;  4) The substrate surface area estimated by the method of aluminum foil wrapping. Area is determined by calculating the area-weight regression curve and weighing the foil. |
| Macrophyte | 1) The quantitative aboveground biomass (wet weigh, g) were quantified by riparian and aquatic plants with 1 m wide × 1 m long quadrats, 2) annual production was calculated by harvest method at the beginning and end in each year (Hauer and Lamberti 2007). |
| Detritus | 1) Fine particular organic matter (FPOM, 0.45 μm < size < 1 mm) were collected by filtering 5-10 L volumes of water through two piled plankton nets (120 μm and 1 mm). Retained fractions were FOMP in the size > 120 μm. The remaining filtered water with FPOM <120 μm were filtered by glass filter (0.45-μm diameter);  2) Organic matters in sediment were calculated as percentage of total organic carbon in total dry matter in the sediment, and the wet weight of organic matters were by obtained dividing the water content in sediment (Carrer and Opitz 1999). |

**Method S3 Conversion factors and empirical formulae used to derive the input parameters of functional groups**

| **Items** | **Conversion factor/formula** | **Reference** |
| --- | --- | --- |
| Fish | Empirical formulae used to calculate *P*/*B* and *Q*/*B* :  *P/B* = *Z* = *K* × (*L*_inf_ – *L*_mean_) / (*L*_mean_ – *L*_c_) for exploited fish;  *P/B* = *M* = *K* ^0.65^ × *L_∞_*^-0.279^ × *T_c_*^0.463^ for non-exploited fish;  Log (*Q/B*) = 7.964 - 0.204 log *W*_inf_ – 1.965 *T* + 0.083 *A* + 0.532 *h* + 0.398 *d*;  *L*_mean_, *L*_c_, *T_c_*, and *T* are measured values, *K*, *L*_inf_, *L_∞_*, *W*_inf_, *A*, and *d* are obtained from FishBase. | Christensen et al. (2008), Froese and Pauly. (2017), Pauly et al. (2000) |
| Invertebrate  (insects, mollusks, decapods, annelids) | 1) Regression of length (mm) – dry weight (g) for aquatic insects; | (Benke et al. 1999) |
|  | 2) Factors used to transform wet weight (g m^-2^) to energy of respiration and production:  1g dry weight ≈ 6 g wet weight ≈ 5 kcal ≈ 0.9 g ash-free dry weight ≈ 0.5 g carbon weight | Morin (1997), Waters (1977) |
|  | 3) Empirical formulae for calculating production (*P*), respiration (*R*), *P*/*B*, and *Q*/*B* :  Log *P* = 0.22 + 1.01 × log *B* – 0.34 × log *M* + 0.037 × *T*;  Log *R* = -0.317 + 0.941 × log *P* | Brey (1999, 2010) |
| Zooplankton | 1) Regression of length (mm) – dry weight (g); | Culver et al. (1985) |
|  | 2) Empirical formulae used to calculate *Q*/*B* is same to that of invertebrate, but for *P*/*B*:  1 g wet weight = 5 g dry weight;  Y/W = R = a × W ^b-1^ a = 10 ^(-2.145+0.037×T)^;  Log (*P/B*) = -0.73 - 0.23 × log (dry weight), CF = 1.12 | Culver et al. (1985)  Leidy and Ploskey (1980)  Kuns and Sprules (2000) |
| Algae | Factors used to transform the phytoplankton chlorophyll a to areal wet weight (g m^-2^):  1 g carbon = 1 g O_2_ × 1/PQ × 12/32; carbon : chlorophyll a = 25:1; 1 g carbon = 3.3 g dry weight; 1 g dry weight = 5.71 g wet weight. | Hauer and Lamberti (2007), Lin et al. (2005) |

**Method S4 Diet composition based on stomach and gut content analysis**

Gravimetric and volumetric methods were combined to quantify the proportion of each prey item in diet composition (*DC*) (Cortés 1997, Hyslop 1980). The stomachs or guts of fish and invertebrates were removed and stored at -18°C. For species that utilize bulky prey, such as nekton, molluscs, hydrophytes and large insects, all the stomach contents were removed and identified; for species that utilize microscopic prey, such as periphyton, plankton or detritus, pre-digested components were selected from the foreguts for detection by microscopy. First, identifiable bulky prey and their fragments were separated under a stereo microscope, leaving a colloidal mixture of unidentifiable contents. The gravimetric method was used to directly measure the wet weight ratio of each selected part and the weight of the unidentified mixture (Baker et al. 2014). Second, the remaining mixture (gastric juice, miniature materials, and tiny prey fragments) was diluted with distilled water (3–5 times the volume of the mixture) and spread onto a modified glass plate, and the components were identified (Hellawell and Abel 1971) under an optical microscope. The volumetric method was used to identify the contents of the plate with a 2 × 2 × 0.01 cm^3^ groove uniformly divided across 10 × 10 cells (2 × 2 × 0.1 mm^3^ for each cell). Five foregut points and 20 cells (2 rows) for each point were randomly selected to estimate the volumetric ratios. Volume calculations were as follows: 1) organic particles, vegetal debris, algae, and protozoans were estimated through approximate geometry; 2) length-volume regression equations were used for microcrustaceans and rotifers; and 3) the amorphous residue of benthos or abiotic items that compacted to fill the plate was estimated from the areas of the cells it covered.

After the fish and invertebrate specimens were collected, the stomach and gut contents were analyzed. Because most aquatic organisms (e.g., fish, shrimp, crab) undergo ontogenetic dietary shifts, fish more than 1 year of age and invertebrates with large body size were selected from the collected specimens for stomach content analysis, and all specimens with empty stomachs were excluded. To avoid cluster sampling, an estimator provided by Latour et al. (2008) and Buchheister and Latour (2015) was used when gravimetrically or volumetrically summarising the per-year *DC* data for each species. The percentage of each prey item in the diet was calculated as its relative contribution to the total stomach or gut contents:

$$\text{GP}_{\text{i}}\text{ (\%) = }\frac{\text{W}_{\text{i}\text{ }}}{\text{W}_{\text{total}}}\text{× 100 (1)}$$

$$\text{VP}_{\text{j}}\text{ (\%) = }\frac{\text{V}_{\text{j }}}{\text{V}_{\text{total}}}\text{ × 100} \text{(2)}$$

$$\text{GP}_{\text{j}}\text{ (\%) = }\frac{\text{W}_{\text{mixture }}}{\text{W}_{\text{total}}}\text{×}\frac{\text{V}_{\text{j }}}{\text{V}_{\text{mixture}}}\text{ × 100 (3)}$$

where *GP_i_* and *GP_j_* are the gravimetric percentages (%) of the bulky prey *i* and the miniature prey *j*, respectively, in the diet; *W_i_*, *W_mixture_*, and *W_total_* are the wet masses of the bulky prey *i*, the remaining mixture, and the total stomach contents, respectively; *VP_j_* is the volumetric percentage (%) of prey *j* in the diet; and *V_j_*, *V_mixture_*, and *V_total_* are the volumes (mm^3^) of the miniature prey *j*, the point of mixture, and the point of the pre-digested contents from the foregut, respectively. Equations (1) and (2) were used for the gravimetric and volumetric approaches, respectively, and Equations (3) was used for the combination of both approaches.

**Appendix B General habitat characteristics and physicochemical parameters of water quality**

**Table S1** General habitat characteristics of the 6 sampling sections along the Pearl River. River sections #1 – #6: agricultural, industrial, island, urban, factory, and estuarine zones.

| Section | #1 | #2 | #3 | #4 | #5 | #6 |
| --- | --- | --- | --- | --- | --- | --- |
| North latitude | 23°9'32.18" | 23°8'14.37" | 23°3'8.18" | 23°3'57.30" | 22°57'40.14" | 22°50'40.60" |
| East longitude | 113°12'40.53" | 113°13'19.01" | 113°19'22.00" | 113°22'32.59" | 113°32'33.70" | 113°34'19.43" |
| Reaches | Lower Liuxi River | Lower Shijing River | Middle Zhujiang River | Lower Zhujiang River | Middle tributary of the lower East River | Human outlet, Pearl River estuary |
| Riparian land use type | Agricultural and rural areas in Baiyun district of Guangzhou | Dense residential population and private workshops near shore, such as catering and leather manufacturing | Island areas in suburb of Guangzhou | Dense urban population in residential areas, interaction of the Front and Back Channel | Ports and factories surrounding the shipping channel, dense rural population near shore | International shipping hubs near the Pearl River Estuary |
| Pollution sources | Aquaculture and rural domestic sewage from adjacent villages and towns | Industrial sewage from the Shijing River | Rural domestic sewage | Point source municipal domestic sewage from Guangzhou | Industrial and domestic wastewater from adjacent towns in Dongguan | Contamination carried by inland rivers and marine oil pollution from ships |
| Elevation (m) | 28.9 | 22.5 | 16.3 | 11.4 | 5.3 | -1.0 |
| Distance to estuary (km) | 81.6 | 68.6 | 54.2 | 41.9 | 28.8 | 16.9 |
| Slope (°) | 18.49 | 13.60 | 8.18 | 4.85 | 1.13 | 0.64 |
| Wetted river width (m) | 244.7 | 327.9 | 586.7 | 643.9 | 1494.4 | 1736.1 |
| Flow discharge (m3/s) | 61.4 | 223.6 | 620.6 | 865.6 | 1104.2 | 1539.7 |
| Riffle habitat (% of habitat area) | 53.24 | 40.26 | 33.14 | 23.07 | 12.46 | 6.95 |
| Pool habitat (% of habitat area) | 18.43 | 12.96 | 9.68 | 8.06 | 6.15 | 0.84 |
| Canopy cover (%) | 43.23 | 33.90 | 20.78 | 10.41 | 5.22 | 2.92 |
| Seasonal floodplain area (m^2^) | 32.83 | 43.76 | 18.46 | 13.21 | 23.23 | 5.12 |
| Riparian buffer width (m) | 8.96 | 4.31 | 3.27 | 0.92 | 3.87 | 2.14 |
| Gravel-cobble substrate (% of substrate area) | 23.26 | 20.39 | 19.31 | 16.53 | 12.62 | 7.63 |
| Silt-sand substrate (% of substrate area) | 35.37 | 40.52 | 49.73 | 65.16 | 87.48 | 100 |
| Urban land (% of land area) | 21.42 | 30.94 | 42.16 | 53.25 | 65.80 | 80.59 |
| Agricultural land (% of land area) | 43.21 | 54.92 | 20.85 | 28.72 | 20.61 | 10.47 |
| Forestry land (% of land area) | 17.73 | 7.14 | 18.63 | 9.18 | 6.84 | 4.60 |
| Riparian vegetation coverage (%) | 53.86 | 43.25 | 37.94 | 30.27 | 15.62 | 7.41 |
| Nearshore concrete revetment (km) | 0.83 | 1.42 | 2.65 | 4.37 | 5.33 | 6.90 |
| Nearshore population (thousand) | 20.3 | 40.4 | 53.3 | 112.1 | 73.6 | 55.3 |

**Table S2** Physicochemical parameters of the water quality along the Pearl River during the wet and dry seasons. Three parallels ("-1", "-2", and "-3") were sampled in each river section. River sections #1 – #6: agricultural, industrial, island, urban, factory, and estuarine zones.

| Section | T  (°C) | | DO  (mg/L) | | EC  (μs/cm) | | TN  (mg/L) | | NO_3_^–^-N  (mg/L) | | NH_4_^+^-N  (mg/L) | | TP  (mg/L) | | COD  (mg/L) | | pH | | Salinity  (‰) | |
| --- | --- | --- | --- | --- | --- | --- | --- | --- | --- | --- | --- | --- | --- | --- | --- | --- | --- | --- | --- | --- |
|  | Wet | Dry | Wet | Dry | Wet | Dry | Wet | Dry | Wet | Dry | Wet | Dry | Wet | Dry | Wet | Dry | Wet | Dry | Wet | Dry |
| #1-1 | 27.3 | 21.3 | 2.83 | 2.78 | 246 | 452 | 3.01 | 5.55 | 1.8 | 2.64 | 0.78 | 0.19 | 0.76 | 0.88 | 44.2 | 37.1 | 7.84 | 7.02 | 0.14 | 0.15 |
| #1-2 | 27.0 | 22.1 | 3.50 | 2.38 | 239 | 495 | 2.79 | 6.44 | 1.86 | 2.20 | 0.67 | 0.21 | 0.25 | 1.16 | 51.5 | 29.6 | 7.68 | 7.10 | 0.19 | 0.18 |
| #1-3 | 28.0 | 20.1 | 3.24 | 2.68 | 221 | 493 | 2.32 | 5.43 | 1.65 | 4.45 | 0.58 | 0.23 | 0.26 | 0.62 | 46.6 | 24.5 | 7.36 | 7.57 | 0.20 | 0.21 |
| #2-1 | 25.2 | 20.9 | 1.50 | 1.58 | 262 | 493 | 2.38 | 5.30 | 1.58 | 4.55 | 0.45 | 0.20 | 0.19 | 1.84 | 49.9 | 25.3 | 7.72 | 7.29 | 0.28 | 0.29 |
| #2-2 | 26.8 | 21.0 | 1.94 | 1.05 | 212 | 484 | 2.39 | 5.29 | 1.43 | 3.55 | 0.72 | 0.24 | 0.19 | 0.52 | 32.5 | 27.1 | 7.22 | 7.45 | 0.30 | 0.32 |
| #2-3 | 25.3 | 20.1 | 1.17 | 0.69 | 281 | 481 | 2.18 | 5.85 | 1.46 | 3.60 | 0.52 | 0.34 | 0.15 | 1.43 | 38.0 | 24.8 | 7.62 | 7.63 | 0.35 | 0.39 |
| #3-1 | 25.5 | 21.9 | 4.64 | 4.42 | 203 | 475 | 2.46 | 5.15 | 1.21 | 2.64 | 0.85 | 0.24 | 0.12 | 0.42 | 28.3 | 25.6 | 7.08 | 7.33 | 0.44 | 0.43 |
| #3-2 | 25.3 | 19.3 | 4.84 | 4.80 | 299 | 468 | 1.98 | 7.14 | 1.33 | 0.47 | 0.58 | 1.89 | 0.11 | 1.02 | 26.1 | 24.2 | 7.51 | 7.96 | 0.47 | 0.55 |
| #3-3 | 25.1 | 22.1 | 5.66 | 4.66 | 126 | 481 | 1.93 | 6.24 | 1.28 | 0.58 | 0.57 | 0.05 | 0.10 | 0.31 | 24.6 | 22.8 | 7.60 | 7.25 | 0.58 | 0.61 |
| #4-1 | 26.1 | 21.9 | 4.06 | 4.80 | 143 | 525 | 1.84 | 6.74 | 1.03 | 0.61 | 0.79 | 1.89 | 0.11 | 0.78 | 22.2 | 25.4 | 7.96 | 7.32 | 0.91 | 0.97 |
| #4-2 | 26.1 | 20.7 | 4.20 | 4.78 | 126 | 811 | 0.93 | 5.62 | 0.55 | 0.09 | 0.36 | 0.02 | 0.06 | 0.62 | 19.0 | 22.6 | 7.04 | 7.53 | 1.39 | 1.24 |
| #4-3 | 26.2 | 19.9 | 4.99 | 4.50 | 107 | 809 | 1.20 | 3.66 | 0.28 | 2.43 | 0.82 | 0.01 | 0.05 | 0.62 | 11.2 | 16.4 | 7.83 | 8.3 | 1.83 | 1.76 |
| #5-1 | 25.4 | 20.6 | 4.08 | 4.72 | 123 | 5854 | 1.89 | 3.34 | 0.05 | 3.14 | 1.64 | 0.05 | 0.04 | 0.15 | 10.7 | 28.3 | 7.14 | 7.87 | 2.54 | 2.89 |
| #5-2 | 25.5 | 20.8 | 4.00 | 4.53 | 268 | 3966 | 1.73 | 2.96 | 0.05 | 1.88 | 1.50 | 0.05 | 0.14 | 0.17 | 11.6 | 25.4 | 7.14 | 7.90 | 2.89 | 2.82 |
| #5-3 | 25.5 | 21.0 | 3.91 | 4.69 | 413 | 2078 | 1.56 | 2.58 | 0.04 | 0.62 | 1.35 | <0.01 | 0.24 | 0.18 | 12.5 | 22.5 | 7.13 | 7.92 | 3.06 | 3.64 |
| #6-1 | 24.8 | 20.4 | 4.82 | 5.90 | 1766 | 2058 | 1.27 | 4.79 | 0.01 | 0.25 | 1.09 | 0.04 | 0.04 | 0.59 | 13.1 | 24.7 | 7.03 | 7.90 | 6.42 | 6.84 |
| #6-2 | 25.6 | 21.4 | 5.68 | 5.10 | 5145 | 6620 | 0.99 | 6.97 | 0.36 | 3.10 | 0.56 | 0.21 | 0.05 | 0.56 | 11.6 | 22.2 | 6.89 | 7.69 | 6.97 | 7.15 |
| #6-3 | 25.7 | 21.4 | 5.21 | 5.15 | 3662 | 5378 | 1.91 | 5.24 | 0.21 | 3.92 | 1.50 | 0.13 | 0.17 | 0.67 | 18.6 | 34.7 | 10.53 | 11.76 | 6.02 | 8.91 |

T, water temperature; DO, dissolved oxygen; EC, electronic conductivity; COD, chemical oxygen demand; TN, total nitrogen; NH_4_^+^-N, ammonia nitrogen; NO_3_^-^-N, nitrate nitrogen TP, total phosphorus.

**Appendix C Input parameters and species composition of the six Ecopath models along the Pearl River**

**Table S3** Basic input parameters for the six Ecopath models, including biomass (*B*), production-to-biomass ratio (*P*/*B*), consumption-to-biomass ratio (*Q*/*B*), unassimilated consumption rate (*UC*).

| **Groups in agricultural zone** | ***B*** | ***P*/*B*** | ***Q*/*B*** | ***UC*** | **Groups in industrial zone** | ***B*** | ***P*/*B*** | ***Q*/*B*** | ***UC*** |
| --- | --- | --- | --- | --- | --- | --- | --- | --- | --- |
|  | g m^-2^ | year^-1^ | year^-1^ |  |  | g m^-2^ | year^-1^ | year^-1^ |  |
| **Fish** |  |  |  |  | **Fish** |  |  |  |  |
| Benthic carnivorous fish | 1.22 | 1.13 | 2.61 | 0.20 | Detritivorous fish | 4.87 | 1.63 | 17.3 | 0.57 |
| Insectivorous fish | 0.04 | 2.40 | 5.14 | 0.26 | **Invertebrate** |  |  |  |  |
| Molluscivorous fish | 0.82 | 3.31 | 7.43 | 0.34 | Oligochaete | 1.72 | 1.12 | 19.3 | 0.53 |
| Omnivorous fish | 1.09 | 4.15 | 9.68 | 0.40 | Gastropod | 0.65 | 1.66 | 21.6 | 0.53 |
| Phytoplanktivorous fish | 0.44 | 2.76 | 13.7 | 0.57 | Decapod larvae | 0.19 | 1.83 | 36.9 | 0.53 |
| Herbivorous fish | 0.09 | 6.76 | 25.4 | 0.60 | Cladoceran | 0.11 | 20.1 | 116 | 0.60 |
| Detritivorous fish | 9.90 | 3.73 | 17.6 | 0.67 | Copepod | 0.18 | 19.8 | 146 | 0.60 |
| **Invertebrate** |  |  |  |  | Rotifer | 0.24 | 15.5 | 242 | 0.60 |
| Aquatic insect larvae | 0.05 | 9.74 | 27.5 | 0.60 | **Producer** |  |  |  |  |
| Shrimp | 0.82 | 10.4 | 25.9 | 0.60 | Phytoplankton | 2.58 | 135 |  |  |
| Crab | 0.44 | 13.9 | 33.5 | 0.64 | Periphyton | 0.40 | 35.9 |  |  |
| Oligochaete | 0.48 | 19.8 | 57.7 | 0.53 | **Detritus** | 126 |  |  |  |
| Gastropod | 5.27 | 23.9 | 62.9 | 0.59 |  |  |  |  |  |
| Bivalve | 2.51 | 30.2 | 85.0 | 0.60 |  |  |  |  |  |
| Copepod | 0.23 | 48.1 | 268 | 0.68 |  |  |  |  |  |
| Cladoceran | 0.30 | 55.7 | 320 | 0.71 |  |  |  |  |  |
| Rotifer | 0.81 | 61.6 | 339 | 0.73 |  |  |  |  |  |
| **Producer** |  |  |  |  |  |  |  |  |  |
| Phytoplankton | 2.47 | 166 |  |  |  |  |  |  |  |
| Periphyton | 0.72 | 146 |  |  |  |  |  |  |  |
| Macrophyte | 4.07 | 6.09 |  |  |  |  |  |  |  |
| **Detritus** | 175 |  |  |  |  |  |  |  |  |

‒ Table S3 Continued ‒

| **Groups in island zone** | ***B*** | ***P*/*B*** | ***Q*/*B*** | ***UC*** | **Groups in urban zone** | ***B*** | ***P*/*B*** | ***Q*/*B*** | ***UC*** |
| --- | --- | --- | --- | --- | --- | --- | --- | --- | --- |
|  | g m^-2^ | year^-1^ | year^-1^ |  |  | g m^-2^ | year^-1^ | year^-1^ |  |
| **Reptiles** |  |  |  |  | **Reptiles** |  |  |  |  |
| Chinese softshell turtle | 0.07 | 0.21 | 6.76 | 0.20 | Chinese softshell turtle | 0.88 | 1.11 | 6.67 | 0.35 |
| **Fish** |  |  |  |  | Red-eared slider | 0.90 | 1.24 | 6.87 | 0.37 |
| Piscivorous fish | 0.35 | 2.13 | 9.61 | 0.20 | **Fish** |  |  |  |  |
| Pelagic carnivorous fish | 0.92 | 2.63 | 13.2 | 0.23 | Benthic carnivorous fish | 1.94 | 1.13 | 4.61 | 0.20 |
| Benthic carnivorous fish | 2.84 | 2.53 | 15.6 | 0.20 | Molluscivorous fish | 1.31 | 2.31 | 5.43 | 0.26 |
| Molluscivorous fish | 1.54 | 3.31 | 16.4 | 0.26 | Omnivorous fish | 4.14 | 1.15 | 12.7 | 0.40 |
| Omnivorous fish | 5.09 | 4.15 | 19.7 | 0.40 | Phytoplanktivorous fish | 2.07 | 2.76 | 24.7 | 0.57 |
| Zooplanktivorous fish | 0.62 | 5.86 | 9.85 | 0.35 | Detritivorous fish | 1.81 | 3.73 | 17.6 | 0.67 |
| Phytoplanktivorous fish | 2.65 | 7.76 | 24.7 | 0.57 | **Invertebrate** |  |  |  |  |
| Herbivorous fish | 0.24 | 6.74 | 23.4 | 0.59 | Shrimp | 1.38 | 10.4 | 25.9 | 0.60 |
| Detritivorous fish | 8.98 | 8.73 | 27.6 | 0.61 | Crab | 1.21 | 13.9 | 33.5 | 0.64 |
| **Invertebrate** |  |  |  |  | Gastropod | 3.72 | 23.9 | 168 | 0.59 |
| Shrimp | 2.51 | 5.67 | 29.9 | 0.58 | Bivalve | 1.47 | 30.2 | 189 | 0.60 |
| Crab | 4.56 | 10.4 | 35.9 | 0.65 | Polychaete | 0.91 | 5.67 | 36.9 | 0.70 |
| Gastropod | 6.41 | 23.9 | 181 | 0.59 | Oligochaete | 0.18 | 19.8 | 152 | 0.53 |
| Bivalve | 2.73 | 40.2 | 198 | 0.60 | Copepod | 0.41 | 48.1 | 285 | 0.68 |
| Polychaete | 0.81 | 5.67 | 76.9 | 0.70 | Cladoceran | 0.23 | 55.7 | 301 | 0.71 |
| Oligochaete | 0.64 | 19.8 | 113.7 | 0.53 | Rotifer | 1.20 | 61.6 | 326 | 0.73 |
| Copepod | 0.25 | 48.1 | 263.3 | 0.68 | **Producer** |  |  |  |  |
| Cladoceran | 0.17 | 60.7 | 300 | 0.71 | Phytoplankton | 9.29 | 201 |  |  |
| Rotifer | 0.86 | 65.6 | 311 | 0.73 | Macrophyte | 1.07 | 3.25 |  |  |
| **Producer** |  |  |  |  | Periphyton | 2.61 | 60.9 |  |  |
| Phytoplankton | 3.33 | 279 |  |  | **Detritus** | 75.0 |  |  |  |
| Periphyton | 0.94 | 196 |  |  |  |  |  |  |  |
| Macrophyte | 2.11 | 10.1 |  |  |  |  |  |  |  |
| **Detritus** | 75.1 |  |  |  |  |  |  |  |  |
|  |  |  |  |  |  |  |  |  |  |
|  |  |  |  |  |  |  |  |  |  |
|  |  |  |  |  |  |  |  |  |  |

‒ Table S3 Continued ‒

| **Groups in factory zone** | ***B*** | ***P*/*B*** | ***Q*/*B*** | ***UC*** | **Groups in estuarine zone** | ***B*** | ***P*/*B*** | ***Q*/*B*** | ***UC*** |
| --- | --- | --- | --- | --- | --- | --- | --- | --- | --- |
|  | g m^-2^ | year^-1^ | year^-1^ |  |  | g m^-2^ | year^-1^ | year^-1^ |  |
| **Fish** |  |  |  |  | **Fish** |  |  |  |  |
| Benthic carnivorous fish | 2.93 | 1.26 | 6.63 | 0.20 | Piscivorous fish | 2.71 | 2.63 | 15.6 | 0.20 |
| Molluscivorous fish | 2.88 | 1.03 | 12.5 | 0.20 | Pelagic carnivorous fish | 3.70 | 4.63 | 18.2 | 0.23 |
| Omnivorous fish | 4.97 | 2.86 | 17.9 | 0.20 | Benthic carnivorous fish | 2.24 | 3.13 | 18.5 | 0.29 |
| Phytoplanktivorous fish | 3.18 | 2.56 | 18.3 | 0.20 | Molluscivorous fish | 2.31 | 2.31 | 19.0 | 0.33 |
| Herbivorous fish | 0.29 | 3.01 | 18.1 | 0.20 | Omnivorous fish | 5.09 | 4.15 | 25.7 | 0.42 |
| Detritivorous fish | 5.17 | 3.92 | 20.6 | 0.20 | Zooplanktivorous fish | 1.25 | 6.76 | 23.8 | 0.35 |
| **Invertebrate** |  |  |  |  | Phytoplanktivorous fish | 3.42 | 6.76 | 21.7 | 0.57 |
| Shrimp | 1.62 | 12.2 | 27.7 | 0.40 | Detritivorous fish | 8.98 | 3.73 | 32.6 | 0.67 |
| Crab | 1.53 | 13.9 | 33.5 | 0.60 | **Invertebrate** |  |  |  |  |
| Polychaete | 1.19 | 9.36 | 89.5 | 0.64 | Omnivorous shrimp | 4.91 | 19.7 | 44.9 | 0.60 |
| Gastropod | 4.24 | 13.5 | 92.3 | 0.40 | Carnivorous shrimp | 1.02 | 14.2 | 36.0 | 0.24 |
| Bivalve | 2.35 | 20.7 | 112 | 0.40 | Crab | 2.22 | 26.9 | 67.9 | 0.59 |
| Copepod | 1.61 | 36.9 | 175 | 0.40 | Gastropod | 1.40 | 15.5 | 108 | 0.68 |
| Cladoceran | 0.40 | 58.6 | 214 | 0.60 | Bivalve | 3.91 | 39.2 | 84.0 | 0.60 |
| Rotifer | 1.88 | 65.2 | 249 | 0.60 | Polychaete | 2.75 | 22.8 | 152 | 0.53 |
| **Producer** |  |  |  |  | Copepod | 3.50 | 48.1 | 225 | 0.68 |
| Phytoplankton | 6.21 | 296 |  |  | Cladoceran | 0.05 | 55.7 | 311 | 0.71 |
| Periphyton | 1.95 | 189 |  |  | Rotifer | 2.25 | 67.6 | 376 | 0.73 |
| **Detritus** | 375 |  |  |  | **Producer** |  |  |  |  |
|  |  |  |  |  | Phytoplankton | 7.03 | 202 |  |  |
|  |  |  |  |  | Periphyton | 6.19 | 48.6 |  |  |
|  |  |  |  |  | **Detritus** | 75.1 |  |  |  |

**Table S4** The percentage (%) of each prey in diet composition of predators in river sections #1 – #6.

| **Prey** | | **Diet composition of predators in agricultural zone #1** | | | | | | | | | | | | | | | |
| --- | --- | --- | --- | --- | --- | --- | --- | --- | --- | --- | --- | --- | --- | --- | --- | --- | --- |
|  |  | 1 | 2 | 3 | 4 | 5 | 6 | 7 | 8 | 9 | 10 | 11 | 12 | 13 | 14 | 15 | 16 |
| 1 | Benthic carnivorous fish | 0.078 |  |  |  |  |  |  |  |  |  |  |  |  |  |  |  |
| 2 | Insectivorous fish | 0.022 |  |  |  |  |  |  |  |  |  |  |  |  |  |  |  |
| 3 | Molluscivorous fish | 0.131 |  |  |  |  |  |  |  |  |  |  |  |  |  |  |  |
| 4 | Omnivorous fish | 0.149 |  |  |  |  |  |  |  |  |  |  |  |  |  |  |  |
| 5 | Phytoplanktivorous fish | 0.040 |  |  |  |  |  |  |  |  |  |  |  |  |  |  |  |
| 6 | Herbivorous fish | 0.001 |  |  |  |  |  |  |  |  |  |  |  |  |  |  |  |
| 7 | Detritivorous fish | 0.232 |  |  |  |  |  |  |  |  |  |  |  |  |  |  |  |
| 8 | Aquatic insect larvae |  | 0.676 |  |  |  |  |  |  |  |  |  |  |  |  |  |  |
| 9 | Shrimp | 0.176 | 0.113 | 0.176 |  |  |  |  |  |  |  |  |  |  |  |  |  |
| 10 | Crab | 0.087 |  |  |  |  |  |  |  |  |  |  |  |  |  |  |  |
| 11 | Oligochaete |  |  |  |  |  |  | 0.051 |  |  |  |  |  |  |  |  |  |
| 12 | Gastropod | 0.083 |  | 0.159 | 0.091 |  |  |  |  |  | 0.062 |  |  |  |  |  |  |
| 13 | Bivalve |  |  | 0.421 | 0.370 |  | 0.063 |  |  |  | 0.017 |  |  |  |  |  |  |
| 14 | Copepod |  |  |  | 0.062 | 0.078 |  | 0.011 |  | 0.129 | 0.127 | 0.001 | 0.003 | 0.010 | 0.002 | 0.003 |  |
| 15 | Cladoceran |  |  |  | 0.013 | 0.016 |  | 0.013 |  | 0.398 | 0.017 | 0.002 | 0.014 | 0.002 | 0.002 | 0.004 |  |
| 16 | Rotifer |  |  |  | 0.025 | 0.009 |  | 0.036 |  | 0.099 | 0.028 | 0.029 | 0.007 | 0.005 | 0.085 | 0.012 | 0.006 |
| 17 | Phytoplankton |  |  |  |  | 0.476 |  |  |  | 0.014 | 0.050 | 0.110 | 0.029 | 0.419 | 0.417 | 0.318 | 0.251 |
| 18 | Periphyton |  | 0.212 |  | 0.022 | 0.134 | 0.014 | 0.046 | 0.162 | 0.137 | 0.015 |  | 0.116 | 0.100 | 0.078 | 0.101 | 0.126 |
| 19 | Macrophyte |  |  |  | 0.127 |  | 0.684 |  |  |  | 0.183 |  |  |  |  |  |  |
| 20 | Detritus |  |  | 0.243 | 0.291 | 0.288 | 0.239 | 0.843 | 0.838 | 0.223 | 0.500 | 0.858 | 0.831 | 0.464 | 0.417 | 0.562 | 0.616 |

-Table S4 Continued-

| **Prey** | | **Diet composition of predators in industrial zone #2** | | | | | | |
| --- | --- | --- | --- | --- | --- | --- | --- | --- |
|  |  | 1 | 2 | 3 | 4 | 5 | 6 | 7 |
| 1 | Detritivorous fish |  |  |  |  |  |  |  |
| 2 | Oligochaete |  |  |  |  |  |  |  |
| 3 | Gastropod | 0.004 |  |  |  |  |  |  |
| 4 | Decapod larvae | 0.001 |  |  |  |  |  |  |
| 5 | Cladoceran | 0.003 | 0.005 | 0.001 | 0.005 |  | 0.002 |  |
| 6 | Copepod | 0.005 | 0.019 |  | 0.151 |  |  |  |
| 7 | Rotifer | 0.016 | 0.023 |  | 0.003 |  | 0.003 |  |
| 8 | Phytoplankton | 0.143 | 0.311 | 0.306 | 0.201 | 0.572 | 0.472 | 0.581 |
| 9 | Periphyton | 0.120 |  | 0.143 | 0.133 | 0.002 | 0.002 | 0.002 |
| 10 | Detritus | 0.707 | 0.642 | 0.550 | 0.507 | 0.426 | 0.521 | 0.417 |

‒ Table S4 Continued ‒

| **Prey** | | **Diet composition of predators in island zone #3** | | | | | | | | | | | | | | | | | | | |
| --- | --- | --- | --- | --- | --- | --- | --- | --- | --- | --- | --- | --- | --- | --- | --- | --- | --- | --- | --- | --- | --- |
|  |  | 1 | 2 | 3 | 4 | 5 | 6 | 7 | 8 | 9 | 10 | 11 | 12 | 13 | 14 | 15 | 16 | 17 | 18 | 19 |  |
| 1 | Chinese softshell turtle | 0.014 |  |  |  |  |  |  |  |  |  |  |  |  |  |  |  |  |  |  |  |
| 2 | Piscivorous fish | 0.317 | 0.089 |  |  |  |  |  |  |  |  |  |  |  |  |  |  |  |  |  |  |
| 3 | Pelagic carnivorous fish | 0.272 | 0.173 | 0.054 | 0.004 |  |  |  |  |  |  |  |  |  |  |  |  |  |  |  |  |
| 4 | Benthic carnivorous fish | 0.163 | 0.156 | 0.043 | 0.042 |  |  |  |  |  |  |  |  |  |  |  |  |  |  |  |  |
| 5 | Molluscivorous fish | 0.009 | 0.030 | 0.067 | 0.066 |  |  |  |  |  |  |  |  |  |  |  |  |  |  |  |  |
| 6 | Omnivorous fish | 0.027 | 0.063 | 0.076 | 0.074 |  |  |  |  |  |  |  |  |  |  |  |  |  |  |  |  |
| 7 | Zooplanktivorous fish | 0.018 | 0.034 | 0.053 | 0.040 |  |  |  |  |  |  |  |  |  |  |  |  |  |  |  |  |
| 8 | Phytoplanktivorous fish | 0.027 | 0.064 | 0.125 | 0.134 |  |  |  |  |  |  |  |  |  |  |  |  |  |  |  |  |
| 9 | Herbivorous fish | 0.014 | 0.005 |  |  |  |  |  |  |  |  |  |  |  |  |  |  |  |  |  |  |
| 10 | Detritivorous fish | 0.045 | 0.153 | 0.483 | 0.519 |  |  |  |  |  |  |  |  |  |  |  |  |  |  |  |  |
| 11 | Shrimp | 0.051 | 0.131 | 0.042 | 0.066 |  |  | 0.068 |  |  |  |  |  |  |  |  |  |  |  |  |  |
| 12 | Crab | 0.043 | 0.101 | 0.051 | 0.051 |  |  |  |  |  |  |  |  |  |  |  |  |  |  |  |  |
| 13 | Gastropod |  |  |  |  | 0.400 | 0.359 |  |  |  |  | 0.094 | 0.079 | 0.008 |  |  |  |  |  |  |  |
| 14 | Bivalve |  |  |  |  | 0.600 | 0.521 |  |  |  |  | 0.056 | 0.047 |  |  |  |  |  |  |  |  |
| 15 | Polychaete |  |  | 0.005 | 0.004 |  |  |  |  |  |  | 0.005 | 0.005 |  |  |  |  |  |  |  |  |
| 16 | Oligochaete |  |  |  |  |  |  |  |  |  |  | 0.027 | 0.023 | 0.050 |  | 0.141 |  |  |  |  |  |
| 17 | Copepod |  |  |  |  |  |  | 0.137 | 0.023 |  |  | 0.001 | 0.001 | 0.001 | 0.001 |  | 0.002 | 0.001 | 0.003 | 0.004 |  |
| 18 | Cladoceran |  |  |  |  |  |  | 0.311 | 0.007 |  |  | 0.003 | 0.002 | 0.002 | 0.002 |  | 0.001 | 0.003 | 0.004 | 0.005 |  |
| 19 | Rotifer |  |  |  |  |  |  | 0.413 | 0.066 |  |  | 0.004 | 0.003 |  | 0.011 |  | 0.003 | 0.006 | 0.013 | 0.015 |  |
| 20 | Phytoplankton |  |  |  |  |  |  |  | 0.685 |  | 0.094 | 0.203 | 0.170 | 0.150 | 0.448 | 0.323 | 0.415 | 0.594 | 0.553 | 0.523 |  |
| 21 | Periphyton |  |  |  |  |  |  |  |  |  | 0.105 | 0.285 | 0.237 |  | 0.120 | 0.051 | 0.053 | 0.088 | 0.100 | 0.075 |  |
| 21 | Macrophyte |  |  |  |  |  | 0.120 |  |  | 0.538 | 0.014 |  |  |  |  |  |  |  |  |  |  |
| 23 | Detritus |  |  |  |  |  |  | 0.070 | 0.219 | 0.462 | 0.787 | 0.321 | 0.434 | 0.789 | 0.418 | 0.485 | 0.526 | 0.309 | 0.329 | 0.379 |  |

‒ Table S4 Continued ‒

| **Prey** | | **Diet composition of predators in urban zone #4** | | | | | | | | | | | | | | | |
| --- | --- | --- | --- | --- | --- | --- | --- | --- | --- | --- | --- | --- | --- | --- | --- | --- | --- |
|  |  | 1 | 2 | 3 | 4 | 5 | 6 | 7 | 8 | 9 | 10 | 11 | 12 | 13 | 14 | 15 | 16 |
| 1 | Chinese softshell turtle |  |  |  |  |  |  |  |  |  |  |  |  |  |  |  |  |
| 2 | Red-eared slider | 0.016 |  |  |  |  |  |  |  |  |  |  |  |  |  |  |  |
| 3 | Benthic carnivorous fish | 0.132 |  | 0.013 |  |  |  |  |  |  |  |  |  |  |  |  |  |
| 4 | Molluscivorous fish | 0.148 |  | 0.116 |  |  |  |  |  |  |  |  |  |  |  |  |  |
| 5 | Omnivorous fish | 0.013 | 0.127 | 0.103 |  |  |  |  |  |  |  |  |  |  |  |  |  |
| 6 | Phytoplanktivorous fish | 0.013 | 0.140 | 0.067 |  |  |  |  |  |  |  |  |  |  |  |  |  |
| 7 | Detritivorous fish | 0.013 | 0.218 | 0.056 |  |  |  |  |  |  |  |  |  |  |  |  |  |
| 8 | Shrimp | 0.163 | 0.014 | 0.089 | 0.050 | 0.042 |  |  |  |  |  |  |  |  |  |  |  |
| 9 | Crab | 0.013 | 0.014 | 0.022 | 0.035 | 0.001 |  |  |  |  |  |  |  |  |  |  |  |
| 10 | Gastropod | 0.150 | 0.041 | 0.260 | 0.307 | 0.444 |  |  |  |  |  |  | 0.029 |  |  |  |  |
| 11 | Bivalve | 0.171 | 0.127 | 0.117 | 0.133 | 0.135 |  |  |  | 0.069 |  |  | 0.061 |  |  |  |  |
| 12 | Polychaete |  |  | 0.065 | 0.324 | 0.139 |  |  |  | 0.019 |  |  | 0.015 |  |  |  |  |
| 13 | Oligochaete |  |  |  | 0.019 | 0.056 |  |  | 0.049 | 0.030 | 0.040 |  | 0.034 |  |  |  |  |
| 14 | Copepod |  |  |  |  |  | 0.001 |  | 0.163 | 0.019 | 0.008 | 0.001 | 0.012 | 0.002 | 0.001 | 0.002 | 0.003 |
| 15 | Cladoceran |  |  |  |  |  | 0.002 |  | 0.165 | 0.031 | 0.002 | 0.002 | 0.014 | 0.001 | 0.002 | 0.003 | 0.004 |
| 16 | Rotifer |  |  |  |  |  | 0.012 |  | 0.023 | 0.056 | 0.100 | 0.009 | 0.015 | 0.003 | 0.005 | 0.010 | 0.012 |
| 17 | Phytoplankton |  |  |  |  |  | 0.514 |  | 0.229 | 0.017 | 0.092 | 0.541 | 0.213 | 0.394 | 0.675 | 0.642 | 0.618 |
| 18 | Macrophyte |  | 0.158 |  | 0.006 |  |  |  |  |  |  |  |  |  |  |  |  |
| 19 | Periphyton |  |  |  |  |  |  |  |  | 0.204 | 0.121 |  | 0.144 | 0.001 | 0.001 | 0.010 | 0.010 |
| 20 | Detritus | 0.167 | 0.162 | 0.092 | 0.126 | 0.182 | 0.471 | 1.000 | 0.372 | 0.556 | 0.637 | 0.447 | 0.463 | 0.599 | 0.316 | 0.333 | 0.353 |

‒ Table S4 Continued ‒

| **Prey** | | **Diet composition of predators in factory zone #5** | | | | | | | | | | | | | |
| --- | --- | --- | --- | --- | --- | --- | --- | --- | --- | --- | --- | --- | --- | --- | --- |
|  |  | 1 | 2 | 3 | 4 | 5 | 6 | 7 | 8 | 9 | 10 | 11 | 12 | 13 | 14 |
| 1 | Benthic carnivorous fish | 0.099 |  |  |  |  |  |  |  |  |  |  |  |  |  |
| 2 | Molluscivorous fish | 0.120 |  |  |  |  |  |  |  |  |  |  |  |  |  |
| 3 | Omnivorous fish | 0.194 |  |  |  |  |  |  |  |  |  |  |  |  |  |
| 4 | Phytoplanktivorous fish | 0.070 |  |  |  |  |  |  |  |  |  |  |  |  |  |
| 5 | Herbivorous fish | 0.020 |  |  |  |  |  |  |  |  |  |  |  |  |  |
| 6 | Detritivorous fish | 0.292 |  |  |  |  |  |  |  |  |  |  |  |  |  |
| 7 | Shrimp |  | 0.189 | 0.024 |  |  |  |  |  | 0.014 |  |  |  |  |  |
| 8 | Crab |  | 0.124 |  |  |  |  |  |  |  |  |  |  |  |  |
| 9 | Polychaete | 0.049 | 0.013 |  |  |  |  |  |  |  |  |  |  |  |  |
| 10 | Gastropod | 0.106 | 0.204 | 0.352 |  |  |  | 0.009 | 0.051 |  |  |  |  |  |  |
| 11 | Bivalve |  | 0.224 | 0.228 |  |  |  | 0.008 | 0.015 |  |  |  |  |  |  |
| 12 | Copepod |  |  |  | 0.001 |  |  | 0.162 | 0.012 | 0.002 | 0.001 | 0.001 | 0.002 | 0.003 |  |
| 13 | Cladoceran |  |  |  | 0.002 |  |  | 0.001 | 0.014 | 0.001 | 0.002 | 0.002 | 0.003 | 0.004 |  |
| 14 | Rotifer |  |  |  | 0.012 |  |  | 0.089 | 0.074 | 0.003 | 0.009 | 0.005 | 0.010 | 0.012 | 0.005 |
| 15 | Phytoplankton |  |  |  | 0.514 |  |  | 0.033 |  | 0.406 | 0.546 | 0.575 | 0.541 | 0.456 | 0.342 |
| 16 | Periphyton |  |  |  |  |  |  | 0.135 | 0.124 | 0.115 |  | 0.050 | 0.001 | 0.101 | 0.101 |
| 17 | Detritus | 0.050 | 0.246 | 0.396 | 0.471 | 1.000 | 1.000 | 0.562 | 0.709 | 0.458 | 0.441 | 0.367 | 0.443 | 0.424 | 0.552 |

‒ Table S4 Continued ‒

| **Prey** | | **Diet composition of predators in estuarine zone #6** | | | | | | | | | | | | | | | | |
| --- | --- | --- | --- | --- | --- | --- | --- | --- | --- | --- | --- | --- | --- | --- | --- | --- | --- | --- |
|  |  | 1 | 2 | 3 | 4 | 5 | 6 | 7 | 8 | 9 | 10 | 11 | 12 | 13 | 14 | 15 | 16 | 17 |
| 1 | Piscivorous fish |  |  |  |  |  |  |  |  |  |  |  |  |  |  |  |  |  |
| 2 | Pelagic carnivorous fish | 0.147 | 0.143 |  |  |  |  |  |  |  |  |  |  |  |  |  |  |  |
| 3 | Benthic carnivorous fish | 0.053 | 0.064 |  |  |  |  |  |  |  |  |  |  |  |  |  |  |  |
| 4 | Molluscivorous fish | 0.020 | 0.013 |  |  |  |  |  |  |  |  |  |  |  |  |  |  |  |
| 5 | Omnivorous fish | 0.072 | 0.033 |  |  |  |  |  |  |  |  |  |  |  |  |  |  |  |
| 6 | Zooplanktivorous fish | 0.036 | 0.059 | 0.026 |  |  |  |  |  |  |  |  |  |  |  |  |  |  |
| 7 | Phytoplanktivorous fish | 0.006 | 0.025 | 0.044 |  |  |  |  |  |  |  |  |  |  |  |  |  |  |
| 8 | Detritivorous fish | 0.222 | 0.115 | 0.144 |  |  |  |  |  |  | 0.024 |  |  |  |  |  |  |  |
| 9 | Omnivorous shrimp | 0.254 | 0.242 | 0.425 | 0.248 | 0.239 |  |  |  |  | 0.048 |  |  |  |  |  |  |  |
| 10 | Carnivorous shrimp | 0.074 | 0.056 | 0.107 |  |  |  |  |  |  | 0.004 |  |  |  |  |  |  |  |
| 11 | Crab | 0.085 | 0.035 | 0.092 | 0.017 | 0.021 |  |  |  |  | 0.700 |  |  |  |  |  |  |  |
| 12 | Gastropod |  |  |  | 0.110 | 0.110 |  |  |  |  | 0.029 | 0.020 |  |  |  |  |  |  |
| 13 | Bivalve | 0.013 | 0.150 | 0.103 | 0.421 | 0.419 |  |  |  | 0.067 | 0.029 | 0.120 |  |  |  |  |  |  |
| 14 | Polychaete |  | 0.065 |  | 0.043 | 0.021 |  |  |  | 0.051 | 0.035 | 0.065 |  |  |  |  |  |  |
| 15 | Copepod | 0.019 |  |  |  |  | 0.141 |  |  |  | 0.003 |  |  | 0.004 |  | 0.003 | 0.002 |  |
| 16 | Cladoceran |  |  |  |  |  | 0.001 |  |  |  |  |  |  | 0.003 |  |  |  |  |
| 17 | Rotifer |  |  |  |  |  | 0.003 |  |  |  |  |  |  | 0.006 |  | 0.104 | 0.007 |  |
| 18 | Phytoplankton |  |  |  |  |  | 0.100 | 0.786 |  | 0.350 |  | 0.275 | 0.043 | 0.151 | 0.441 | 0.398 | 0.298 | 0.654 |
| 19 | Periphyton |  |  |  |  |  |  |  |  | 0.031 |  |  | 0.141 |  |  | 0.054 | 0.054 |  |
| 20 | Detritus |  |  | 0.058 | 0.161 | 0.190 | 0.755 | 0.214 | 1.000 | 0.501 | 0.128 | 0.520 | 0.816 | 0.836 | 0.559 | 0.441 | 0.639 | 0.346 |

**Table S5** Details of the fish samples collected along the Pearl River

| **Order/Family/Species** | **Body length**  **(cm)** | **Body weight**  **(g)** | **Age (year)** | **Major food sources** | **Living environment** |
| --- | --- | --- | --- | --- | --- |
|  |  |  |  |  |  |
| **Cypriniformes** |  |  |  |  |  |
| Cyprinidae |  |  |  |  |  |
| *Cirrhinus molitorella* | 15.1-24.6 | 71-151 | 1.5-2.5 | Depositional particles on substrate | Fresh water, demersal |
| *Cirrhinus mrigala* | 15.7-25.5 | 102-167 | 1.5-2.0 | Depositional particles on substrate | Fresh water, demersal |
| *Labeo rohita* |  |  |  | Depositional particles on substrate | Fresh water, demersal |
| *Carassius auratus* | 9.8-14.7 | 57-107.5 | 1.0-1.5 | Depositional particles on substrate | Fresh water, demersal |
| *Hemiculter leucisculus* | 12.2-19.9 | 21-35 | 0.5-1.5 | Suspended particulates in water | Fresh water, pelagic |
| *Hemiculterella wui* |  |  |  | Suspended particulates in water | Fresh water, pelagic |
| *Pseudolaubuca sinensis* |  |  |  | Suspended particulates in water | Fresh water, pelagic |
| *Cyprinus carpio* | 23.4-41.0 | 1019-4064 | 1.5-3.0 | Molluscs (bivalves) | Fresh water, demersal |
| *Squaliobarbus curriculus* | 15.1-20.7 | 113-184 | 1.0-2.0 | Molluscs (bivalves) | Fresh water, demersal |
| *Parabramis pekinensis* | 17.5-24.2 | 72-141 | 1.0-1.5 | Molluscs (bivalves) | Fresh water, demersal |
| *Sinibrama macrops* |  |  |  | Molluscs, hydrophytes | Fresh water, demersal |
| *Hypophthalmichthys molitrix* | 22.6-36.8 | 1646-2164 | 1.5-2.5 | Plankton and fine particles in water | Fresh water, pelagic |
| *Aristichthys nobilis* | 26.4-47.8 | 593-4477 | 2.0-3.5 | Plankton and fine particles in water | Fresh water, pelagic |
| *Ctenopharyngodon idellus* | 21.8-38.6 | 225-990 | 1.0-3.0 | Hydrophytes | Fresh water, demersal |
| *Elopichthys bambusa* | 45.0 | 3451 | 2.0-3.0 | Shrimp, fish | Fresh water, pelagic |
| *Erythroculter reeurviceps* | 26.7-31.4 | 141-230 | 1.5-2.5 | Shrimp, fish | Fresh water, pelagic |
| Cobitidae |  |  |  |  |  |
| *Misgurnus anguillicaudatus* | 15.9-17.2 | 13-16 | 1.0-2.0 | Organic sediment and detritus | Freshwater, demersal |
| *Paramisgurnus dabryanus* |  |  |  | Organic sediment and detritus | Freshwater, demersal |
| **Characiformes** |  |  |  |  |  |
| Curimatidae |  |  |  |  |  |
| *Prochilodus lineatus* |  |  |  | Organic sediment and detritus | Freshwater, demersal |
| Characidae |  |  |  |  |  |
| *Piaractus brachypomus* |  |  |  | Fish | Fresh water, pelagic |
| **Perciformes** |  |  |  |  |  |
| Cichlidae |  |  |  |  |  |
| *Tilapia zillii* | 15.5-18.6 | 97-143 | 1.0-2.0 | Depositional particles on substrate | Freshwater, demersal |
| *Oreochromis niloticus* |  |  |  | Depositional particles on substrate | Freshwater, demersal |
| Leiognathidae |  |  |  |  |  |
| *Leiognathus brevirostris* |  |  |  | Organic sediment and detritus | Brackish water, pelagic |
| Anabantidae |  |  |  |  |  |
| *Anabas testudineus* |  |  |  | Insects, worms, detritus | Freshwater, demersal |
| Channidae |  |  |  |  |  |
| *Channa maculate* | 20.8-29.5 | 79-419 | 1.5-2.5 | Shrimp, fish | Freshwater, demersal |
| *Channa asiatica* |  |  |  | Shrimp, fish, insects | Freshwater, demersal |
| Lateolabracidae |  |  |  |  |  |
| *Lateolabrax maculatus* | 17.5-36.4 | 178-2677 | 1.5-3.0 | Fish, shrimp | Brackish water, demersal |
| Sciaenidae |  |  |  |  |  |
| *Collichthys lucidus* | 16.8-17.9 | 156-254 | 1.0-2.0 | Shrimp, fish, zooplankton | Brackish water, pelagic |
| *Nibea albiflora* |  |  |  | Organic sediment and detritus | Brackish water, pelagic |
| Taenioididae |  |  |  |  |  |
| *Odontamblyopus rubicundus* | 7.5-11 | 14-16 | 1.0-1.5 | Nereids, shrimp, fish | Brackish water, demersal |
| *Taenioides cirratus* |  |  |  | Nereids, shrimp, fish | Brackish water, demersal |
| Gobiidae |  |  |  |  |  |
| *Chaeturichthys stigmatias* |  |  |  | Nereids, shrimp, fish | Brackish water, demersal |
| *Trypauchen vagina* |  |  |  | Nereids, shrimp, fish | Brackish water, demersal |
| *Glossogobiuss giuris* | 7.1-15.8 | 9-32 | 1.0-2.0 | Shrimp, fish, Nereids, gammarids | Brackish water, demersal |
| *Tridentiger barbatus* |  |  |  | Shrimp, fish, Nereids, gammarids | Brackish water, demersal |
| *Boleophthalmus pectinirostris* |  |  |  | Crabs, Nereids, insects | Brackish water, surficial |
| Eleotridae |  |  |  |  |  |
| *Eleotris oxycephala* |  |  |  | Shrimp, fish | Freshwater, demersal |
| Sparidae |  |  |  |  |  |
| *Acanthopagrus latus* |  |  |  | Molluscs (bivalves) | Brackish water, demersal |
| **Siluriformes** |  |  |  |  |  |
| Loricariidae |  |  |  |  |  |
| *Hypostomus plecostomus* | 27.2-29.8 | 203-239 | 1.0-3.0 | Organic sediment and detritus | Freshwater, demersal |
| Clariidae |  |  |  |  |  |
| *Clarias lazera* | 29.1-41.2 | 156-627 | 1.5-2.5 | Shrimp, fish | Freshwater, demersal |
| *Clarias fuscus* |  |  |  | Shrimp, fish | Freshwater, demersal |
| Siluridae |  |  |  |  |  |
| *Silurus cochinchinensis* |  |  |  | Shrimp, fish | Freshwater, demersal |
| Ariidae |  |  |  |  |  |
| *Arius sinensis* | 9.2-17.8 | 37-98 | 1.0-2.0 | Shrimp, crabs, fish | Brackish water, demersal |
| Bagridae |  |  |  |  |  |
| *Pelteobagrus fulvidraco* |  |  |  | Shrimp, fish | Freshwater, demersal |
| Pangasidae |  |  |  |  |  |
| *Pangasius sutchi* |  |  |  | Organic sediment and detritus | Freshwater, demersal |
| **Clupeiformes** |  |  |  |  |  |
| Clupeidae |  |  |  |  |  |
| *Clupanodon thrissa* | 9.0-14.6 | 35.1-57.1 | 0.5-1.5 | Plankton and fine particles in water | Brackish water, pelagic |
| Engraulidae |  |  |  |  |  |
| *Coilia grayi* | 4.9-7.9 | 20.0-32.5 | 1.0-1.5 | Zooplankton, shrimp, fish | Brackish water, pelagic |
| *Thryssa mystax* |  |  |  | Plankton and fine particles in water | Brackish water, pelagic |
| **Mugiliformes** |  |  |  |  |  |
| Mugilidae |  |  |  |  |  |
| *Mugil cephalus* | 18.7-25.9 | 108-289 | 1.0-2.5 | Organic sediment and detritus | Brackish water, demersal |
| **Synbgranchiformes** |  |  |  |  |  |
| Synbranchidae |  |  |  |  |  |
| *Monopterus albus* |  |  |  | Molluscs, insects, shrimp | Freshwater, demersal |
| **Anguilliformes** |  |  |  |  |  |
| Anguillidae |  |  |  |  |  |
| *Anguilla japonica* |  |  |  | Shrimp, fish | Brackish water, demersal |
| Muraenesocidae |  |  |  |  |  |
| *Muraenesox cinereus* | 20.8-33.8 | 88-177 | 1.5-2.5 | Shrimp, fish | Brackish water, demersal |
| **Scorpaeniformes** |  |  |  |  |  |
| Platycephalidae |  |  |  |  |  |
| *Platycephalus indicus* |  |  |  | Molluscs, decapod larvae | Brackish water, demersal |
| **Pleuronectiformes** |  |  |  |  |  |
| Cynoglossidae |  |  |  |  |  |
| *Cynoglossus joyneri* | 10.1-14.6 | 26-67 | 1.0-2.0 | Molluscs, decapod larvae | Brackish water, demersal |
| **Tetraodontiformes** |  |  |  |  |  |
| Tetraodontidae |  |  |  |  |  |
| *Takifugu ocellatus* |  |  |  | Molluscs, decapod larvae | Brackish water, demersal |
| *Takifugu xanthopterus* |  |  |  | Molluscs, decapod larvae | Brackish water, demersal |

**Supplemental Reference**

Baker, R., Buckland, A. and Sheaves, M. (2014) Fish gut content analysis: robust measures of diet composition. Fish and Fisheries 15(1), 170-177.

Benke, A.C., Huryn, A.D., Smock, L.A. and Wallace, J.B. (1999) Length-Mass relationships for freshwater macroinvertebrates in North America with particular reference to the southeastern United States. Journal of the North American Benthological Society 18(3), 308-343.

Bott, T.L., Brock, J.T., Cushing, C.E., Gregory, S.V., King, D. and Petersen, R.C. (1978) A comparison of methods for measuring primary productivity and community respiration in streams. Hydrobiologia 60(1), 3-12.

Brey, T. (1999) A collection of empirical relations for use in ecological modelling. Naga the ICLARM quarterly 22, 24-28.

Brey, T. (2010) An empirical model for estimating aquatic invertebrate respiration. Methods in Ecology and Evolution 1(1), 92-101.

Buchheister, A. and Latour, R.J. (2015) Diets and trophic‐guild structure of a diverse fish assemblage in Chesapeake Bay, U.S.A. Journal of Fish Biology 86(3), 967-992.

Carrer, S. and Opitz, S. (1999) Trophic network model of a shallow water area in the northern part of the Lagoon of Venice. 124(1999), 193-219.

Christensen, V., Walters, C., Pauly, D. and Forrest, R. (2008) Ecopath with Ecosim version 6 User Guide, Fisheries Centre, University of British Columbia, Vancouver, Canada.

Cortés, E. (1997) A critical review of methods of studying fish feeding based on analysis of stomach contents: application to elasmobranch fishes. Canadian Journal of Fisheries and Aquatic Sciences 54(3), 726-738.

Culver, D.A., Boucherle, M.M., Bean, D.J. and Fletcher, J.W. (1985) Biomass of freshwater crustacean zooplankton from length-weight regressions. Canadian Journal of Fisheries and Aquatic Sciences 42(8), 1380-1390.

Froese, R. and Pauly., D. (2017) FishBase, www.fishbase.org, World Wide Web electronic publication.

Hauer, F.R. and Lamberti, G.A. (2007) Methods in Stream Ecology, Academic Press, London.

Hellawell, J. and Abel, R. (1971) A rapid volumetric method for the analysis of the food of fishes. Journal of Fish Biology 3(1), 29-37.

Hyslop, E. (1980) Stomach contents analysis-a review of methods and their application. Journal of Fish Biology 17(4), 411-429.

Kuns, M.M. and Sprules, W.G. (2000) Zooplankton production in Lake Ontario: a multistrata approach. Canadian Journal of Fisheries and Aquatic Sciences 57(11), 2240-2247.

Latour, R.J., Gartland, J., Bonzek, C.F. and Johnson, R.A. (2008) The trophic dynamics of summer flounder (Paralichthys dentatus) in Chesapeake Bay. Fishery Bulletin 106(1), 47-57.

Leidy, G.R. and Ploskey, G.R. (1980) Simulation modeling of zooplankton and benthos in reservoirs: documentation and development of model constructs, Fayetteville, Arkansas.

Lin, H.J., Wang, T.C., Su, H.M. and Hung, J.J. (2005) Relative importance of phytoplankton and periphyton on oyster-culture pens in a eutrophic tropical lagoon. Aquaculture 243(1), 279-290.

Morin, A. (1997) Empirical models predicting population abundance and productivity in lotic systems. Journal of the North American Benthological Society 16(2), 319-337.

Pauly, D., Christensen, V. and Walters, C. (2000) Ecopath, Ecosim, and Ecospace as tools for evaluating ecosystem impact of fisheries. ICES Journal of Marine Science 57(3), 697-706.

SEPA (2002) Standard Methods for Water and Wastewater Monitoring and Analysis, China Environmental Science Press, Beijing.

Waters, T.F. (1977) Advances in Ecological Research. Macfayden, A. and Ford, E.D. (eds), pp. 91-164, Academic Press, London.
